# Real-time scratch assay reveals mechanisms of early calcium signaling in breast cancer cells in response to wounding

**DOI:** 10.18632/oncotarget.25186

**Published:** 2018-05-18

**Authors:** Stephen J.P. Pratt, Erick O. Hernández-Ochoa, Rachel M. Lee, Eleanor C. Ory, James S. Lyons, Humberto C. Joca, Ashley Johnson, Keyata Thompson, Patrick Bailey, Cornell J. Lee, Trevor Mathias, Michele I. Vitolo, Matt Trudeau, Joseph P. Stains, Christopher W. Ward, Martin F. Schneider, Stuart S. Martin

**Affiliations:** ^1^ Department of Biochemistry and Molecular Biology, University of Maryland School of Medicine, Baltimore, MD, USA; ^2^ Department of Physiology, University of Maryland School of Medicine, Baltimore, MD, USA; ^3^ Marlene and Stewart Greenebaum NCI Comprehensive Cancer Center, University of Maryland School of Medicine, Baltimore, MD, USA; ^4^ Department of Orthopaedics, University of Maryland School of Medicine, Baltimore, MD, USA; ^5^ School of Nursing, University of Maryland, Baltimore, MD, USA

**Keywords:** human breast cancer, calcium, mechanotransduction, purinergic receptor, intercellular signaling

## Abstract

Aggressive cellular phenotypes such as uncontrolled proliferation and increased migration capacity engender cellular transformation, malignancy and metastasis. While genetic mutations are undisputed drivers of cancer initiation and progression, it is increasingly accepted that external factors are also playing a major role. Two recently studied modulators of breast cancer are changes in the cellular mechanical microenvironment and alterations in calcium homeostasis. While many studies investigate these factors separately in breast cancer cells, very few do so in combination. This current work sets a foundation to explore mechano-calcium relationships driving malignant progression in breast cancer. Utilizing real-time imaging of an *in vitro* scratch assay, we were able to resolve mechanically-sensitive calcium signaling in human breast cancer cells. We observed rapid initiation of intracellular calcium elevations within seconds in cells at the immediate wound edge, followed by a time-dependent increase in calcium in cells at distances up to 500μm from the scratch wound. Calcium signaling to neighboring cells away from the wound edge returned to baseline within seconds. Calcium elevations at the wound edge however, persisted for up to 50 minutes. Rigorous quantification showed that extracellular calcium was necessary for persistent calcium elevation at the wound edge, but intercellular signal propagation was dependent on internal calcium stores. In addition, intercellular signaling required extracellular ATP and activation of P2Y_2_ receptors. Through comparison of scratch-induced signaling from multiple cell lines, we report drastic reductions in response from aggressively tumorigenic and metastatic cells. The real-time scratch assay established here provides quantitative data on the molecular mechanisms that support rapid scratch-induced calcium signaling in breast cancer cells. These mechanisms now provide a clear framework for investigating which short-term calcium signals promote long-term changes in cancer cell biology.

## INTRODUCTION

Altered calcium handling and the mechanical properties of tumors are both emerging as possible modulators of breast cancer. Tumor rigidity and extracellular matrix (ECM) stiffness are increasingly recognized as important contributors to disease progression [1–4]. Clinically, increasing mammary density is associated with risk for tumor formation [5, 6]. Preclinical studies show that ECM stiffness can disrupt adherens junctions [7], alter cellular differentiation [8], and increase cell proliferation [9] and migration [10]. Interestingly many of these malignant cell phenotypes are also associated with altered calcium signaling [11, 12]. There is growing evidence supporting the notion that calcium signaling is affected at multiple levels in cancer and that calcium, calcium permeable channels, and calcium-binding proteins may play an important role in tumor progression [13–16]. Moreover, epithelial-to-mesenchymal transition (EMT), an activated embryonic program thought to be a central driver in breast cancer malignancy, could be regulated by both matrix mechanics [2, 17] and calcium [18]. There may be a critical intersection between calcium signaling and tumor mechanics in driving aggressive cellular phenotypes, however the precise link remains unclear.

The hypothesis that cancer is a wound that won't heal has historical roots and has been well supported [19]. Indeed there are many similarities between the wounding response and mechanisms that underpin cancer progression. For example, the tumor microenvironment contains many striking parallels to injury associated sites of inflammation including the presence of infiltrating cells, cytokines, and angiogenesis [20, 21]. Likewise, cytoskeletal reorganization, loss of cell-cell contacts, migration and proliferation are necessary steps in both wound re-epithelialization and tumor invasion [19]. It is perhaps not surprising that an *in vitro* wound-surrogate, the scratch-assay, is widely used to study cancer cell signaling and behavior. The scratch assay has also been used to study calcium signaling in non-malignant cell types [22–24]. This provides a unique opportunity to simultaneously investigate early mechanically-stimulated changes in calcium followed by downstream signaling cascades and resulting biological responses such as migration, proliferation and cell-cell communication.

Here we describe early signaling mechanisms in human breast cancer cells in response to mechanical wounding. We were able to resolve mechanically-stimulated calcium signaling at the wound edge and the resulting intercellular communication to distant cells using a real-time scratch assay. Propagation of calcium signaling to distant cells resolved within seconds, while cells at the wound edge demonstrated persistent elevation of calcium for up to 50 minutes. Extracellular calcium was necessary for persistence at the wound edge, but intercellular signaling was dependent on internal calcium stores. Moreover, intercellular signaling required extracellular ATP and activation of P2Y_2_ receptors. Calcium, a ubiquitous second messenger, is involved in many cellular processes identified as hallmarks of cancer such as regulation of the cell cycle, invasion, migration and cell death [25, 26]. By first experimentally defining rapid mechanically-induced calcium signaling in cancer cells, this work sets a foundation to explore mechano-calcium relationships driving malignant progression.

## RESULTS

### MCF-7 cancer cells exhibit mechanically-sensitive calcium signaling

**Figure 1:**
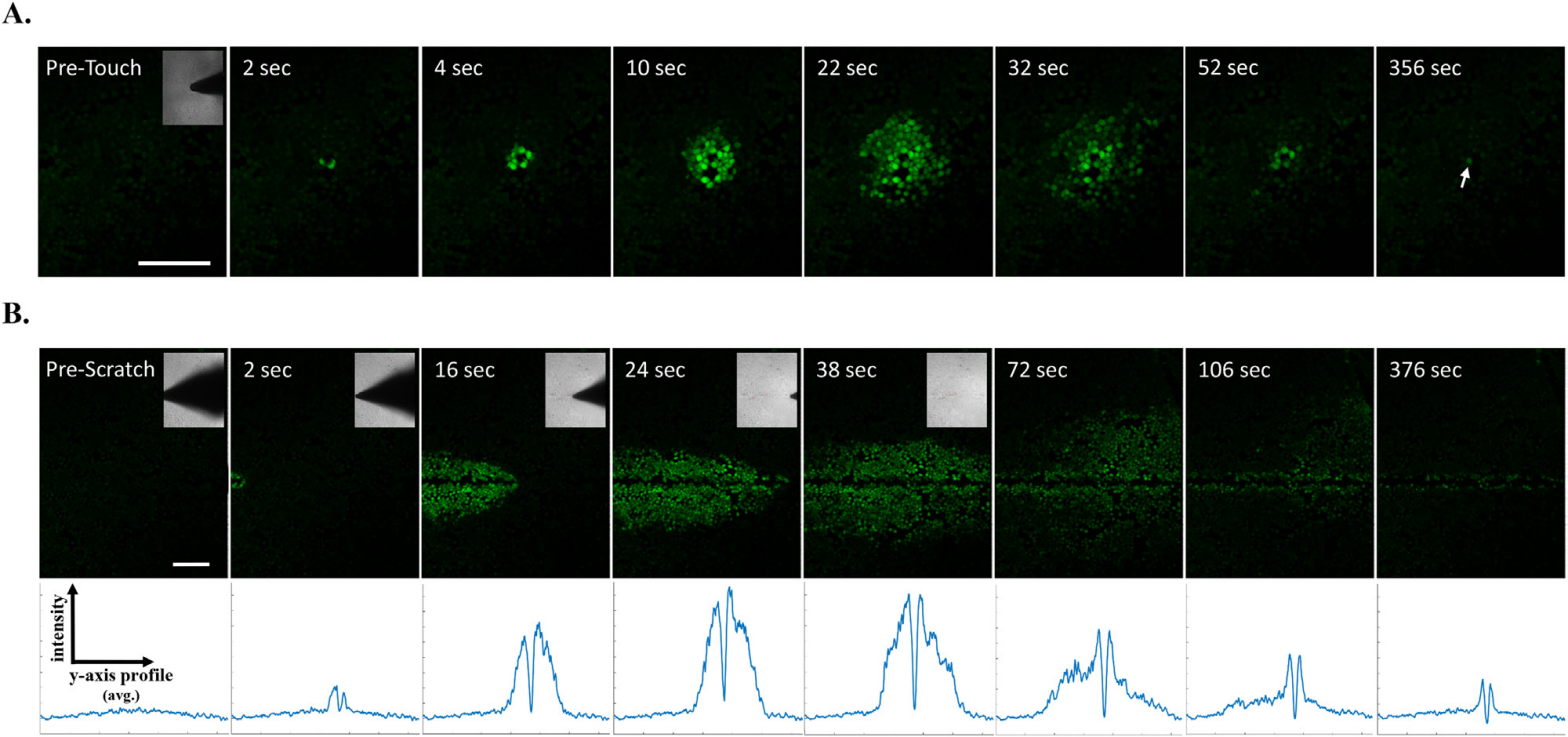
Real-time scratch assay reveals early calcium signaling in MCF-7 cells in response to wounding **(A)** Human breast MCF-7 cancer cells were loaded with the calcium sensitive dye Fluo-4 and mechanically stimulated using a blunt fire-polished glass microprobe. Time series imaging shows that mechanically-induced increases in intracellular calcium occurred in cells that were directly stimulated and was followed by rapid changes in calcium from neighboring cells. This wave-like signal propagation occurred in radial fashion (up to 10 cells away, ~200μm) and was relatively transient compared with a directly stimulated cell showing persistent cytosolic calcium (arrow). **(B)** Fluo-4 loaded MCF-7 cell monolayers were simultaneously scratched with a glass pipette and imaged for 400 seconds (6.7 minutes). Similar to mechanical touch, this real-time scratch assay revealed mechanically-induced increases in intracellular calcium at the wound edge followed by a time-dependent signal propagation to neighboring cells at far distances (~30 cells away, ~500μm). Y-axis *vs.* intensity traces represent data from corresponding x-y frames (positioned directly above). Pixel intensity was plotted for all y-axis points (i.e. 512 pixels or 1.3mm image height) from a single frame, however these values were derived from averaging the pixel intensity across all x-axis points (i.e. direction of scratch for all 512 pixels). These assays indicate intercellular communication in response to changes in the mechanical environment or to wounding. Scale bars equal 200μm.

Mechanically-induced calcium signaling has been established in many epithelial cell types [27, 28] including mammary epithelial cells [29], however the mechanical induction of calcium has not been well characterized in cancer. Earlier reports from mouse mammary tumor cell lines [30–33] showed that mechanical touch can result in rapid calcium signaling across a cell monolayer, and we observe a similar mechanical touch response in MCF-7 breast cancer cells (Figure 1A, Supplementary Video 1). Since scratch wound assays are commonly used in cancer biology to study collective cellular signaling and function (e.g. motility), we decided to examine whether mechanically-induced calcium signals would rapidly result from a scratch wound in MCF-7 breast tumor cells. Confocal time-lapse imaging coupled with a motor-controlled scratch apparatus was used to visualize cancer cell monolayers loaded with the fluorescent calcium indicator, Fluo-4. This real-time scratch assay yields the ability to resolve very early and rapid mechano-signaling events such as calcium signaling. Indeed, increases in intracellular calcium were observed immediately in cells that were directly stimulated by the scratch pipette (Figure 1B, Supplementary Video 2). This was followed by a time-dependent increase in intracellular calcium in cells at much greater distances from the wound edge compared to mechanical touch. This ‘wave-like’ signal propagation across the cell monolayer was apparent from average line traces across the wound (Figure 1B, lower panels). These line traces also showed the wave-like signal was transient compared with persistent calcium signaling in cells at the wound edge, where the line trace retained peaks over time due to calcium activation from wound edge cells (Figure 1B, lower right panel).

**Figure 2:**
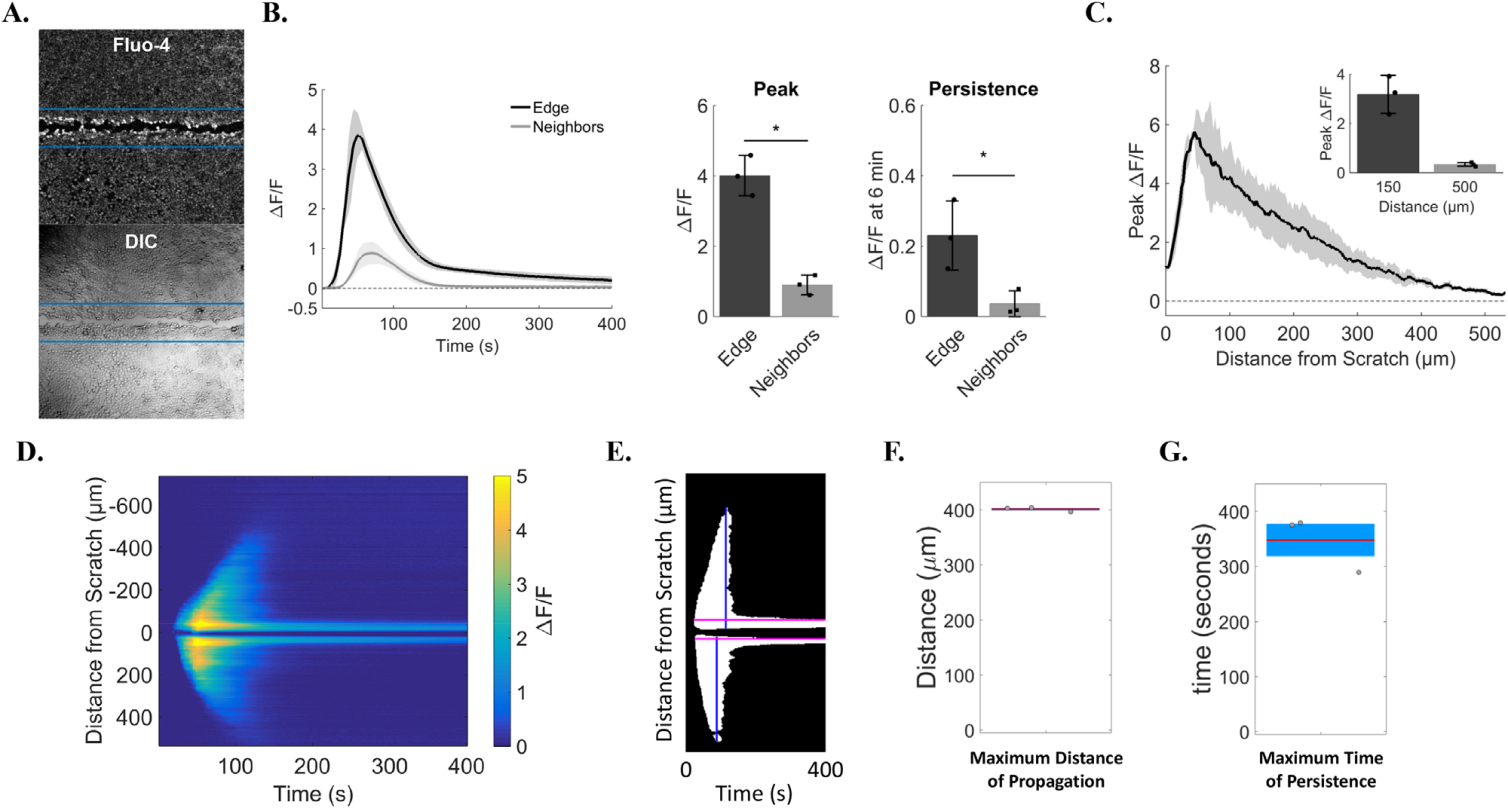
Quantification identifies cell sub-populations with distinct calcium transients **(A)** Relative change in Fluo-4 fluorescence was calculated as ΔF/F using automated regions of interest (ROI). ROI dimensions were set at 1.3mm (image width) by 250μm (125μm on either side of the scratch center line), and were used to quantify changes in calcium from cells at the wound edge (pixels within ROI) *vs.* neighboring cells (remaining pixels in image). Lower panel shows corresponding transmitted light image of upper panel. **(B)** *Left*, traces show ΔF/F data from wound edge cells and neighboring cells over time. *Right*, bar plots summarizing differences in peak and persistent scratch-induced calcium signals between edge and neighboring cells. Edge cells had a larger peak (4.0 ± 0.6) than neighbors (0.9 ± 0.3) and calcium persistence at 6 min was also greater in edge cells (0.2 ± 0.1) compared to neighbors (0.03 ± 0.04). **(C)** Peak ΔF/F was plotted for distances up to 500μm from the edge of the scratch to show total distance of signal propagation to neighboring cells away from the wound edge. Peak ΔF/F values for distances at 150μm and 500μm from the edge are also shown in the bar graph inset. Peak ΔF/F was greatest close to the wound edge (3.2 ± 0.8 at 150μm) *vs.* far distances (0.3 ± 0.1 at 500μm). **(D)** Total distance of signal propagation was also calculated using ROI-independent approaches. Kymographs were generated using y-axis time projections at each time point through 400 seconds. Kymographs show signal intensity propagating away from the wound edge between 40 and 150 seconds which reaches near baseline thereafter. In contrast, signal intensity at the wound edge shows persistence at 400 seconds. **(E)** Kymographs were then converted to binary images using a custom MATLAB based program, from which total time of persistence (in seconds, pink lines) and total distance of signal propagation from the wound edge were quantified (in μm, blue lines). **(F)** Maximum distance of scratch-induced signal propagation to neighboring cells (400.9 ± 3.9μm). **(G)** Maximum time of scratch-induced persistence in cells at the wound edge (347.7 ± 50.8sec). Data presented as mean ± standard deviation. ^*^indicates significance from edge, P < 0.05 via paired t-test. All data represent N=3.

This assay reveals early wound-dependent calcium signaling, which results in a dual-response between cells at the wound edge and signaling to neighboring cells. To rigorously quantitate this phenomenon, we used two approaches to analyze both cell populations. We began with a region of interest (ROI)-based approach (Figure 2A). Automated ROIs were centered on the scratch wound, identified using a custom ‘tip-finding’ software program that uses the pipette tip to independently identify the scratch wound centerline, and encompassed the wound edge using pre-determined dimensions [1.3mm (image width) by 250μm (125μm on either side of the scratch center line)]. Relative fluorescence intensity (ΔF/F) of the calcium indicator Fluo-4 from wound edge ROIs were compared to ΔF/F from the remaining cells (Figure 2A). This approach quantitatively identified differing calcium signals from cells at the wound edge vs neighboring cells (Figure 2B). Wound edge cells not only show greater peak calcium compared with neighboring cells (4.0 ± 0.6 *vs.* 0.9 ± 0.3 ΔF/F, respectively), but also a lasting increase in intracellular calcium (0.2 ± 0.1 in edge *vs.* 0.03 ± 0.04 neighbors, ΔF/F at 6 minutes), as shown in the traces and bar graphs. In addition, manually-selected ROIs were also used to measure ΔF/F between edge *vs.* neighbors and confirm results from automated the ROI-based approach (Supplementary Figures 2 and 3). The total distance of signal propagation from the wound edge to neighboring cells was quantified and shows that, on average, cells up to 500μm away initiated calcium signaling in response to wounding (0.3 ± 0.1 ΔF/F at 500μm, Figure 2C). We also developed a second approach to analyze scratch wound calcium signaling that was ROI-independent. Time projection kymographs of Fluo-4 intensity line averages across the wound were generated for each time point and converted to binary images (Figure 2D, 2E), from which distance from the wound (max distance 400.9 ± 3.9μm) and persistent fluorescence (total persistence 347.7 ± 50.8sec) were measured (Figure 2F, 2G).

### Persistent calcium signaling is not resolved at the edge of a wounded cell monolayer

**Figure 3:**
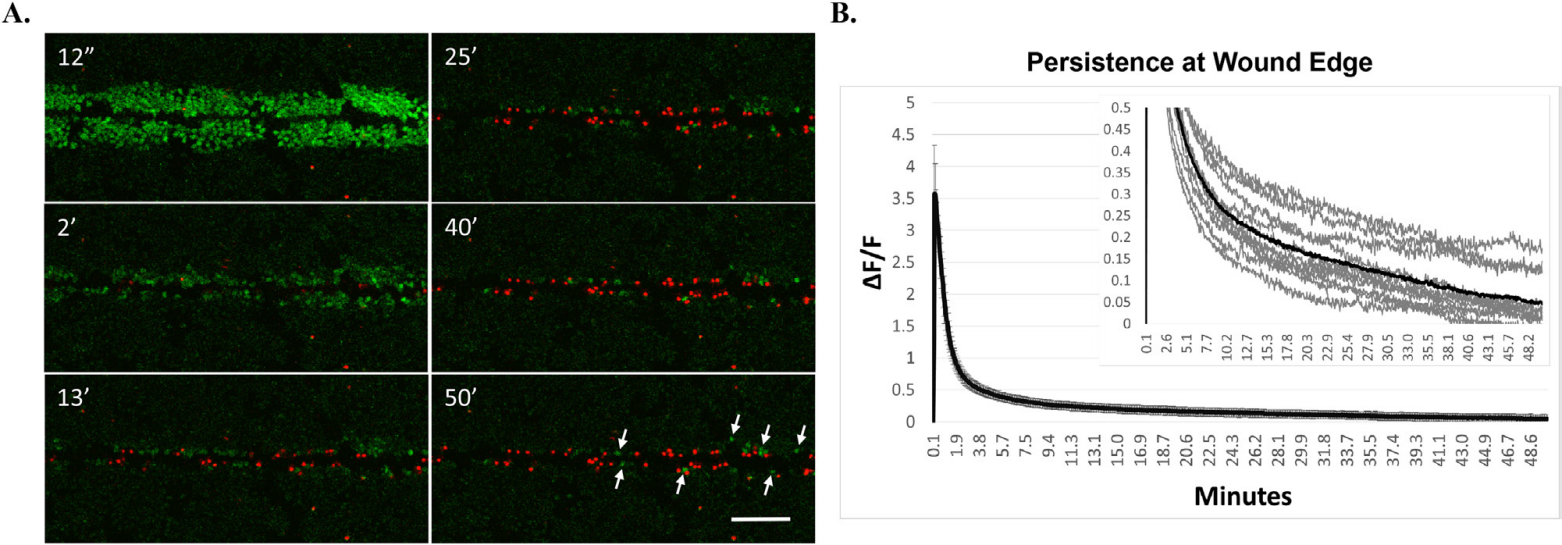
Cells at wound edge show persistent intracellular calcium and remain viable **(A)** MCF-7 cells were loaded with Fluo-4, and propidium iodide (PI, a membrane impermeable nuclear stain) was added to the extracellular media. Cells were scratched and imaged for up to 50 minutes to measure long-term persistence of calcium. Images show that scratch-induced increases in intracellular calcium can persist for long periods of time in cells at the wound edge. While many cells at the scratch wound were PI-positive (indicative of membrane damage), those showing long term persistence were not positive for PI, suggesting that cells at the wound edge can remain viable despite showing persistently elevated calcium (arrows). Scale bar equals 200μm. **(B)** Long term persistence at the wound edge was quantified using manually-generated regions of interest set at the wound edge. Data represent ΔF/F for each time point in a 50 minute time series from 11 biological replicates, across 3 independent experiments. Graph inset shows each individual trace for all biological replicates (gray lines) as well as the total average ΔF/F (black lines) set to different y-axis scaling in order to visualize total persistence (ΔF/F > 0).

Considering the rapidly transient calcium signaling from more canonical cell-types such as muscle and neurons, the persistent calcium signaling up to 6 minutes long in the wound edge cells was surprising and we postulated that it would eventually reach baseline. To this end, we measured scratch-induced calcium signaling and persistence in cell monolayers for up to 50 minutes (Figure 3A, 3B). We additionally identified the presence of dying cells using the fluorescent DNA stain, propidium iodide (PI) which cannot traverse intact membranes, and therefore identifies damaged cells. Wounding resulted in PI positive cells in the immediate wound area (Figure 3A, 2 minute time point). There were also cells that initially showed persistent calcium but became PI positive over time. Astonishingly, there was persistent calcium signaling in PI negative cells at the wound edge after 50 minutes (Figure 3A, arrows). Long-term measurements of unscratched negative control fields indicated no significant increase in Fluo-4 signal over these extended time courses by laser-induced dye activation, establishing that the Fluo-4 signals are induced by the scratch wound itself (Supplementary Figure 1). Quantification from 11 different biological replicates (Figure 3B, inset) shows residual persistent calcium (ΔF/F > 0) at long time periods on average (black trace) and in most individual traces (gray traces). These data indicate that many cells die in response to scratch but it is possible for remaining cells with long periods of elevated cytosolic calcium to maintain viability, at least for the period of time measured here (50 minutes).

### Wound edge *vs* intercellular signaling events require distinct calcium sources

**Figure 4:**
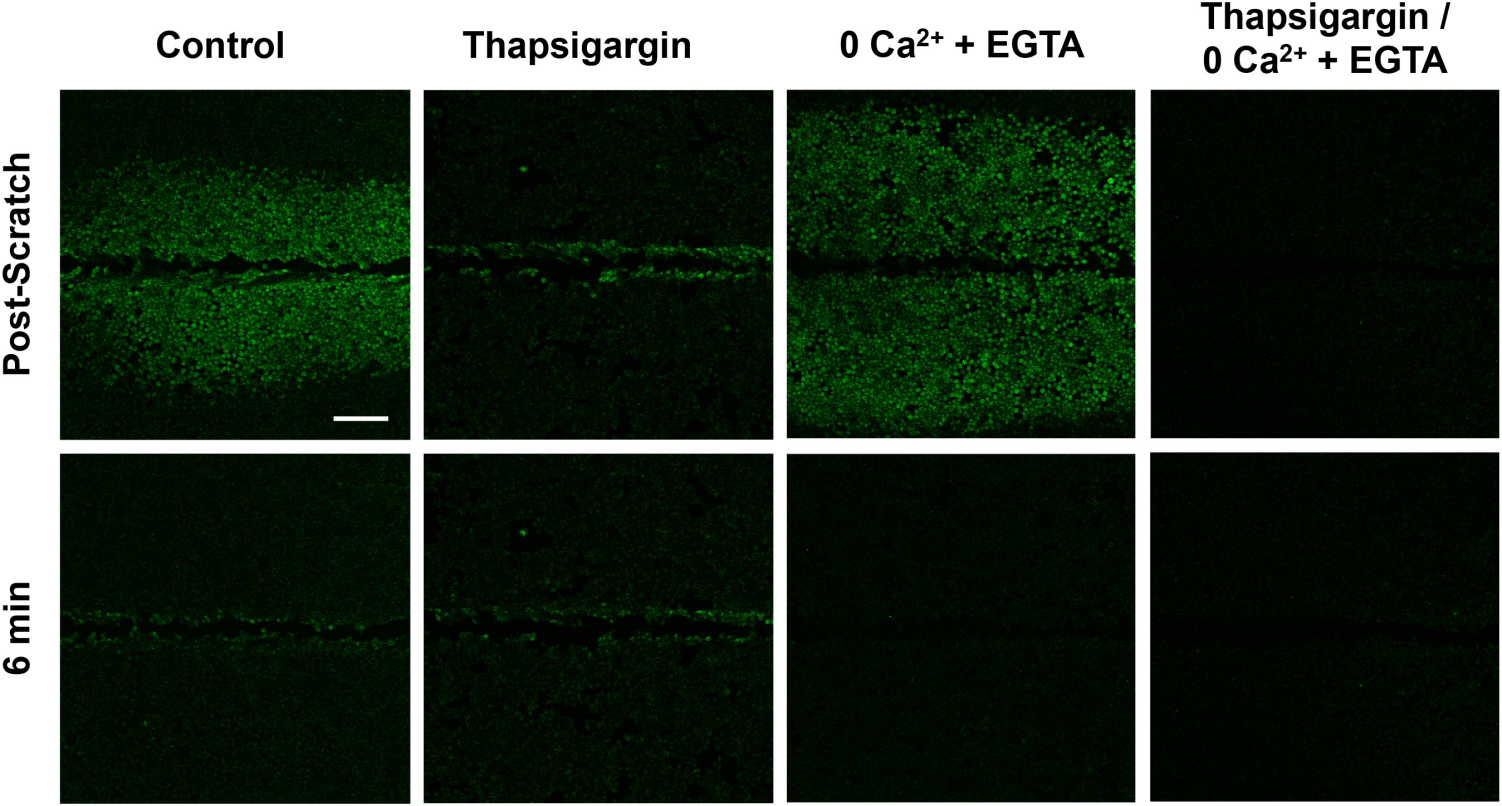
Signal propagation and persistence depend on distinct calcium stores Control groups showed scratch-induced increases in intracellular calcium at the wound edge that persist (measured up to 6 min), followed by propagating and transient calcium in neighboring cells (post-scratch *vs.* 6 min). Compared with control, intracellular calcium depleted (Thapsigargin) cells failed to propagate calcium signaling away from the wound edge. In contrast, depletion of extracellular calcium stores (0Ca^2+^ + EGTA) did result in signal propagation to neighboring cells but blocked persistent calcium at the wound edge. Depletion of both intracellular and extracellular calcium (Thapsigargin/0Ca^2+^ + EGTA) entirely blocked calcium signaling. Scale bar equals 200μm.

**Figure 5:**
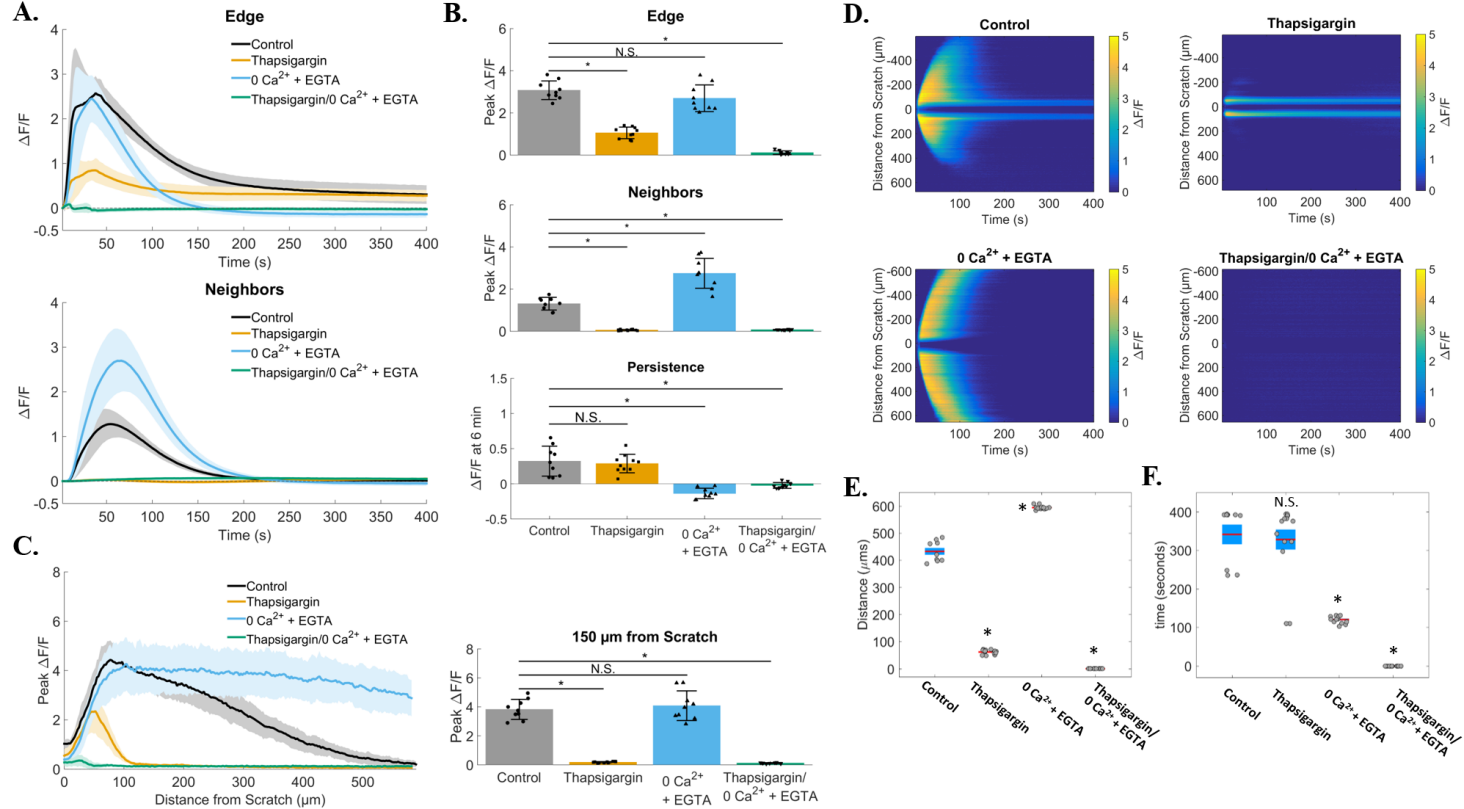
Quantification of zero calcium and thapsigargin treated groups **(A)** Traces show ΔF/F plotted through time for wound edge cells (*upper traces*) *vs.* neighboring cells (*lower traces*) in control, thapsigargin, 0Ca^2+^ + EGTA, and thapsigargin/0Ca^2+^ + EGTA treated groups. **(B)** Summary of peak ΔF/F analysis from wound edge cells and neighboring cells, and persistent calcium signaling at 6 minutes, for each condition tested. Compared with control, peak ΔF/F was blocked in cell neighbors from groups treated with thapsigargin (1.3 ± 0.3 *vs.* 0.06 ± 0.03 respectively), but not with depletion of external calcium (0Ca^2+^ + EGTA, 1.3 ± 0.3 *vs.* 2.7 ± 0.7 respectively). Although calcium signaling occurred in 0Ca^2+^ + EGTA treated edge cells, persistent calcium was completely blocked when compared with control (0.3 ± 0.2 *vs.* −0.1 ± 0.1 respectively). **(C)** Total distance of signal propagation away from the wound edge was plotted for each treatment group. Traces and bar graph show that signaling was preserved at the wound edge with thapsigargin, but propagation away from the edge was blocked [0.2 ± 0.1 at 150μm in thapsigargin *vs.* control (3.8 ± 0.7)]. **(D)** Kymographs (ROI-independent analysis) reveal that signal propagation is blocked with thapisgargin but remains with removal of external calcium (0Ca^2+^ + EGTA), while treatment with 0Ca^2+^ + EGTA blocked persistent calcium at the wound edge. **(E)** Maximum distance was calculated from kymographs for each group and parallel ROI-based analysis (control: 432.4 ± 38.2μm, thapsigargin: 61.8 ± 7.6μm, 0Ca^2+^ + EGTA: 593.7 ± 6.6μm, thapsigargin/0Ca^2+^ + EGTA: 0 ± 0μm). **(F)** Total calcium persistence from kymographs was also quantified and show that persistent calcium was inhibited in 0Ca^2+^ + EGTA treated groups (120.4 ± 9.1sec) compared with control (341.2 ± 76.3sec) and thapsigargin (327.9 ± 97.3). Data presented as mean ± standard deviation. ^*^indicates significance from control, P < 0.05 via one-way ANOVA with a post-hoc Tukey's honest difference criterion. N.S. indicates no significant difference. Data represent N=9 in total from 3 independent experiments for each group.

We set out to first broadly characterize the mechanisms of calcium signaling in the two cell populations (wound-edge *vs.* neighbors) by investigating the dependence of each on intracellular *vs.* extracellular calcium stores (Figure 4). Cells were treated with thapsigargin (2μM x 10 minutes) to deplete intracellular calcium stores in the endoplasmic reticulum (ER). Thapsigargin binds to the sarco-endoplasmic calcium ATPase (SERCA) which prevents calcium re-uptake to the ER. Signal propagation to neighboring cells in response to scratch was blocked in thapsigargin-treated cells (Supplementary Video 3), suggesting that this signaling event requires internal calcium. We then incubated cells in calcium-depleted media (0Ca^2+^ + EGTA) to test the role of external calcium. Cells treated with 0Ca^2+^ + EGTA showed both scratch-induced calcium at the wound edge and propagation to neighboring cells, but blocked persistent calcium at the edge (Supplementary Video 4). As predicted, cells treated with both thapsigargin and 0Ca^2+^ + EGTA completely blocked all calcium signaling (Supplementary Video 5), excluding non-ER sources of intracellular calcium or background Fluo-4 signal. Persistence and propagation to neighbors was quantified (Figure 5) providing detailed data that were consistent with the qualitative observations. Compiled ΔF/F time course data from a total of 36 individual experiments showed that either internal or external calcium was sufficient to trigger calcium signaling at the wound edge, while internal calcium stores were necessary for signaling to neighboring cells and external calcium was necessary for persistence (Figure 5A). Calculations of peak values for edge and neighbors showed that the peak was significantly reduced by thapsigargin for both edge and neighbors, while persistence was significantly affected by 0Ca^2+^ + EGTA (Figure 5B). Interestingly, 0Ca^2+^ + EGTA extended the distance of calcium wave transmission to neighboring cells (Figure 5C). The mechanism for this effect is unknown, however depletion of external Ca^2+^ seems to enhance intercellular communication, possibly through increased ATP release and/or gap junction communication. Kymographs clearly demonstrate the differential effects of thapsigargin on neighboring cell wave formation and 0Ca^2+^ + EGTA on wound edge persistence (Figure 5D). Kymograph-derived measurements of wave distance reinforced conclusions about the inhibitory effect of thapsigargin, as well as the ability of 0Ca^2+^ + EGTA to significantly increase wave transmission distance (Figure 5E). Persistence time was unaffected by thapsigargin, but significantly inhibited by zero calcium (Figure 5F).

### Persistent calcium arises from constitutively active plasma membrane calcium flux

**Figure 6:**
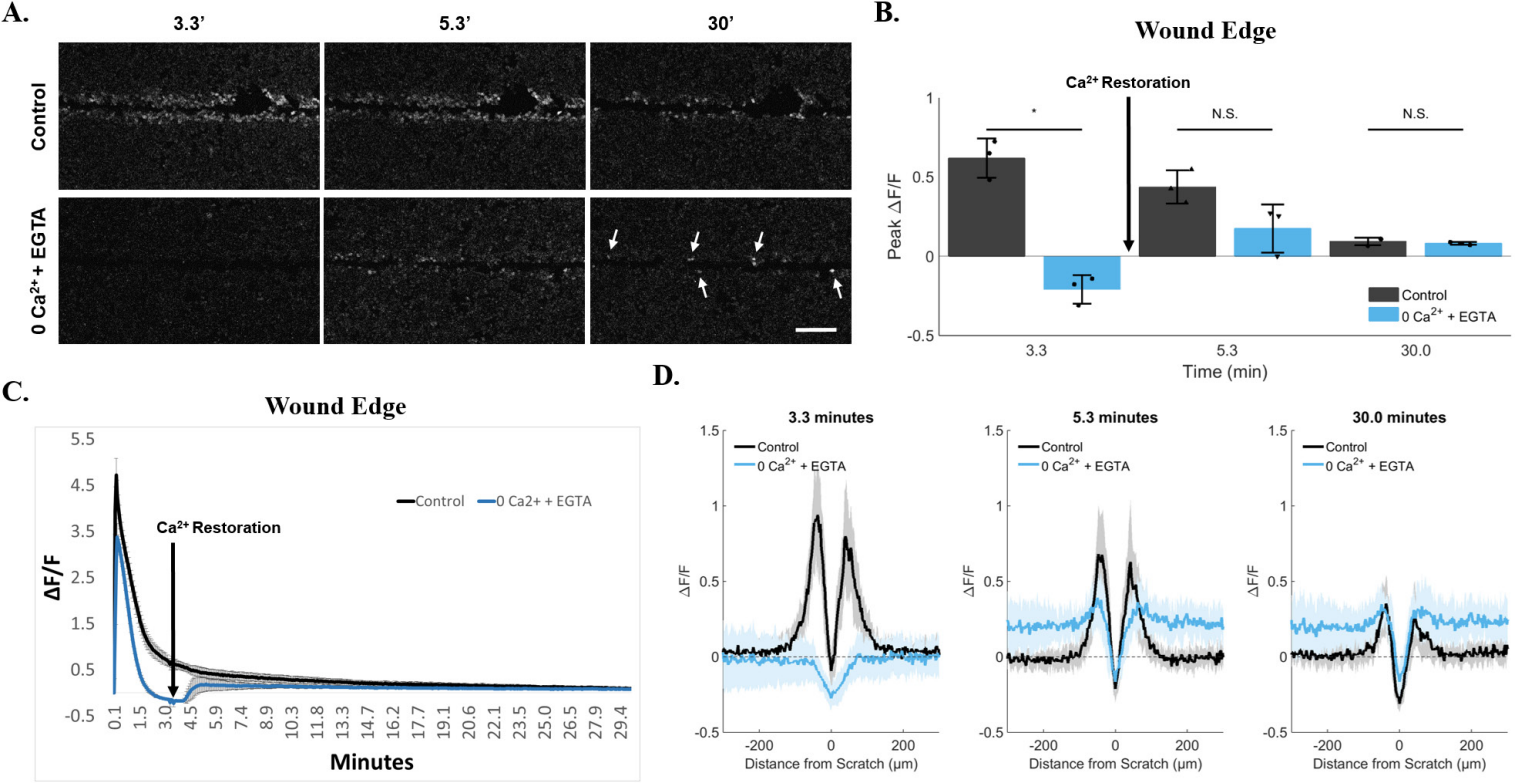
Persistence at the wound edge is regenerated by calcium restoration **(A)** Cells were incubated in 0Ca^2+^ + EGTA media to deplete external calcium and imaged for 30 minutes. Compared with control, persistent calcium was clearly blocked by 200 seconds (3.3 minutes). Calcium was then replenished in the external media via media exchange initiated at 200 seconds (3.3 minutes). This resulted in a sudden and lasting rescue of persistent calcium in cells at the wound edge (arrows). Data was quantified using both ROI-dependent **(B, C)** and ROI-independent approaches **(D)**. Importantly, quantification shows a differential peak ΔF/F between cells at the wound edge *vs.* neighboring cells (D, 0Ca^2+^ + EGTA at 30 minutes). Data presented as mean ± standard deviation. ^*^indicates significance from control, P < 0.05 via paired t-test. For ROI-based analysis (B, C), data represent N=3 [control: 0.6 ± 0.12 at 3.3min, 0.4 ± 0.11 at 5.3min, 0.09 ± 0.02 at 30min. 0Ca2+ + EGTA: −0.2 ± 0.09 at 3.3min, 0.18 ± 0.15 at 5.3min, 0.08 ± 0.01 at 30min]. For ROI-independent analysis (D), data represent N=9 in total from 3 independent experiments. Scale bar equals 200μm.

We further investigated the calcium response at the wound edge using a rescue experiment to replenish calcium after removal from the external media. We hypothesized that in an early and terminal signaling event, persistent calcium would result from external calcium flux across the plasma membrane via calcium permeable channel(s) that could be inhibited by calcium removal but not rescued by calcium restoration during a media exchange. An alternative hypothesis predicted that persistent calcium due to a constitutively active flux of calcium ions across the plasma membrane could be inhibited but then rescued with calcium restoration. Indeed this alternative hypothesis was supported in our rescue experiments where calcium removal initially blocked calcium signaling at the wound edge, when compared with fluorescent wound edge cells in control groups (Figure 6A, 3.3 minutes), but was slowly restored to persistent calcium after media exchange with calcium containing media (Figure 6A, 30 minutes, arrows) (Supplementary Video 6). Thus, a sustained source of external calcium is necessary for persistence at the wound edge. Quantification showed that while peak ΔF/F was significantly different at 3.3 minutes between control cells and cells treated with 0Ca^2+^ + EGTA (Control, 0.6 ± 0.12 *vs.* 0Ca^2+^ + EGTA, −0.2 ± 0.09), immediate restoration of calcium thereafter (media exchange within 3.3 - 5.3 minute period) rendered the wound edge cells similar to the control cells for persistent signal. Once external calcium was restored, there was no apparent significant difference between conditions at either 5.3 minutes (0.4 ± 0.11 *vs.* 0.18 ± 0.15) or 30 minutes (0.09 ± 0.02 *vs.* 0.08 ± 0.01) (Figure 6B). Time course trace data of ΔF/F also highlights the rapid return of Fluo-4 signal at the wound edge upon restoration of calcium (Figure 6C, arrow). Importantly, this increase in ΔF/F after calcium restoration is not simply a phenomenon across the cell population in response to depletion of external calcium, but is also specific to cells at the wound edge (visualized as a differential peak between values close to scratch *vs.* values at greater distances, Figure 6D).

### Intercellular communication is mediated by extracellular ATP and P2Y_2_ purinergic receptor

**Figure 7:**
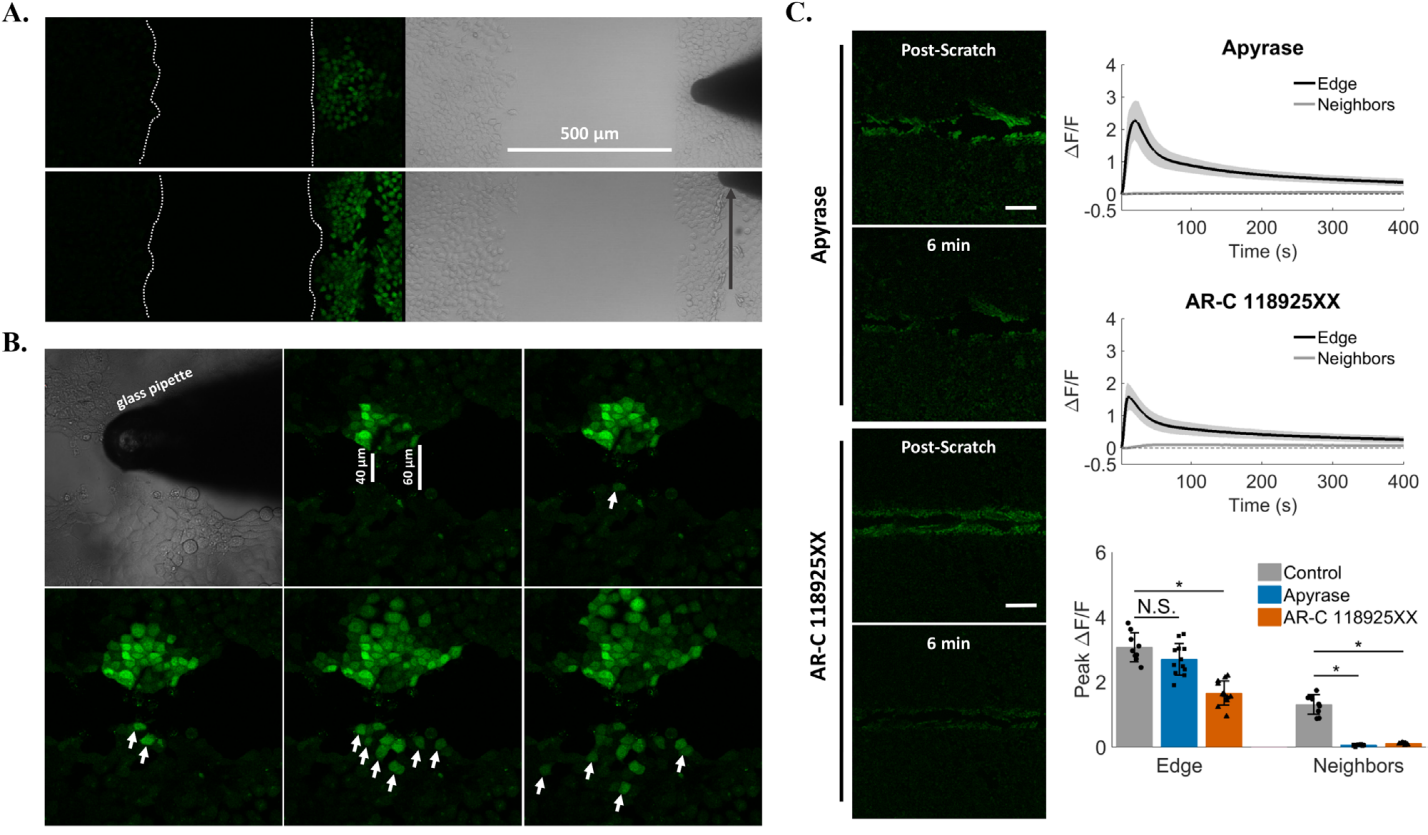
Signal propagation is mediated by extracellular ATP and P2Y_2_ **(A)** Cells were plated in isolation such that two confluent populations were separated by a 500μm gap. Both mechanical touch (top) and scratch (bottom) failed to excite cells across this large gap. **(B)** A smaller separation (40-60 μm) between cells was then generated by scratching a cell monolayer from end to end, and cells were mechanically stimulated on one side of the scratch wound. This resulted in a progressive increase in the number of cells excited across the gap (arrows) suggesting that a cell junction-independent, external signal mediated the mechanically-induced increases in intracellular calcium to neighboring cells. **(C)** Cells were treated with Apyrase for 10 minutes in order to cleave extracellular ATP to ADP/AMP (*top panels*, Apyrase). Scratch resulted in calcium signaling at the wound edge (Peak ΔF/F 2.7 ± 0.5), but Apyrase blocked signal propagation to neighboring cells (Peak ΔF/F 0.06 ± 0.02). Cells were then treated with a selective and competitive P2Y_2_ receptor antagonist (*bottom panels*, AR-C 118925XX), which also blocked signal propagation to neighboring cells after scratch (AR-C 118925XX peak ΔF/F 0.1 ± 0.02 compared with Control peak ΔF/F 1.7 ± 0.4). Data presented as mean ± standard deviation. ^*^indicates significance from control, P < 0.05 via one-way ANOVA with a post-hoc Tukey's honest difference criterion. N.S. indicates no significant difference. Data represent N=12 in total from 3 independent experiments for each group.

To investigate a role for a soluble external signal in our wounding system, we tested if propagation of calcium across a monolayer was dependent on cell-to-cell contact. Cells were first plated into two confluent populations separated by a 500μm gap. Mechanical touch of cells on one side did not result in signal propagation across the gap to the other side. Moreover cell scratch, a more robust mechanical stimulus that can transmit a calcium signal at least 500μm to neighboring cells, also did not produce signal propagation to cells across the gap (Figure 7A). It is possible that any potential soluble signal was unable to travel longer distances due to instability or degradation. We then created a smaller 40-60μm gap by imposing a scratch wound to a cell monolayer from end to end and tested if mechanical touch produced signal propagation (Figure 7B). Indeed, mechanically-stimulated cells on one side of the smaller scratch resulted in a progressive increase in the number of cells on the other side showing intracellular calcium signaling (Figure 7B, arrows). These results suggest that an external, cell-to-cell contact independent signaling molecule could be mediating the signal propagation to neighboring cells in response to wounding.

In response to mechanical stress [34] and injury [35], cells can release the intercellular signaling molecule ATP. We therefore used the enzyme Apyrase in the external media to cleave extracellular ATP to ADP/AMP and measured scratch-induced signaling propagation to cells away from the wound edge (Figure 7C, Supplementary Figure 4). Treatment with Apyrase resulted in a complete block of calcium signaling in neighboring cells (peak ΔF/F 0.06 ± 0.02 compared with edge peak ΔF/F 2.7 ± 0.5) (Supplementary Video 7). If the signal propagation was dependent on extracellular ATP, then by logical extension a plasma membrane receptor activated by ATP such as a purinergic receptor was a likely candidate acceptor of the extracellular ATP signal. In fact expression of certain P2Y family purinergic receptors have been reported in epithelial cells [36], including P2Y_2_ in MCF-7 cells [37]. We next used a pharmacologic approach to block the ATP/UTP activated P2Y_2_ receptor (Figure 7C, Supplementary Figure 4). Like Apyrase, the specific P2Y_2_ receptor antagonist AR-C-118925XX [38] completely blocked scratch-induced signaling to neighboring cells away from the wound edge (peak ΔF/F 0.1 ± 0.02 compared with edge peak ΔF/F 1.7 ± 0.4) (Supplementary Video 8).

### Signaling in response to scratch is disrupted in multiple breast cancer cell lines

**Figure 8:**
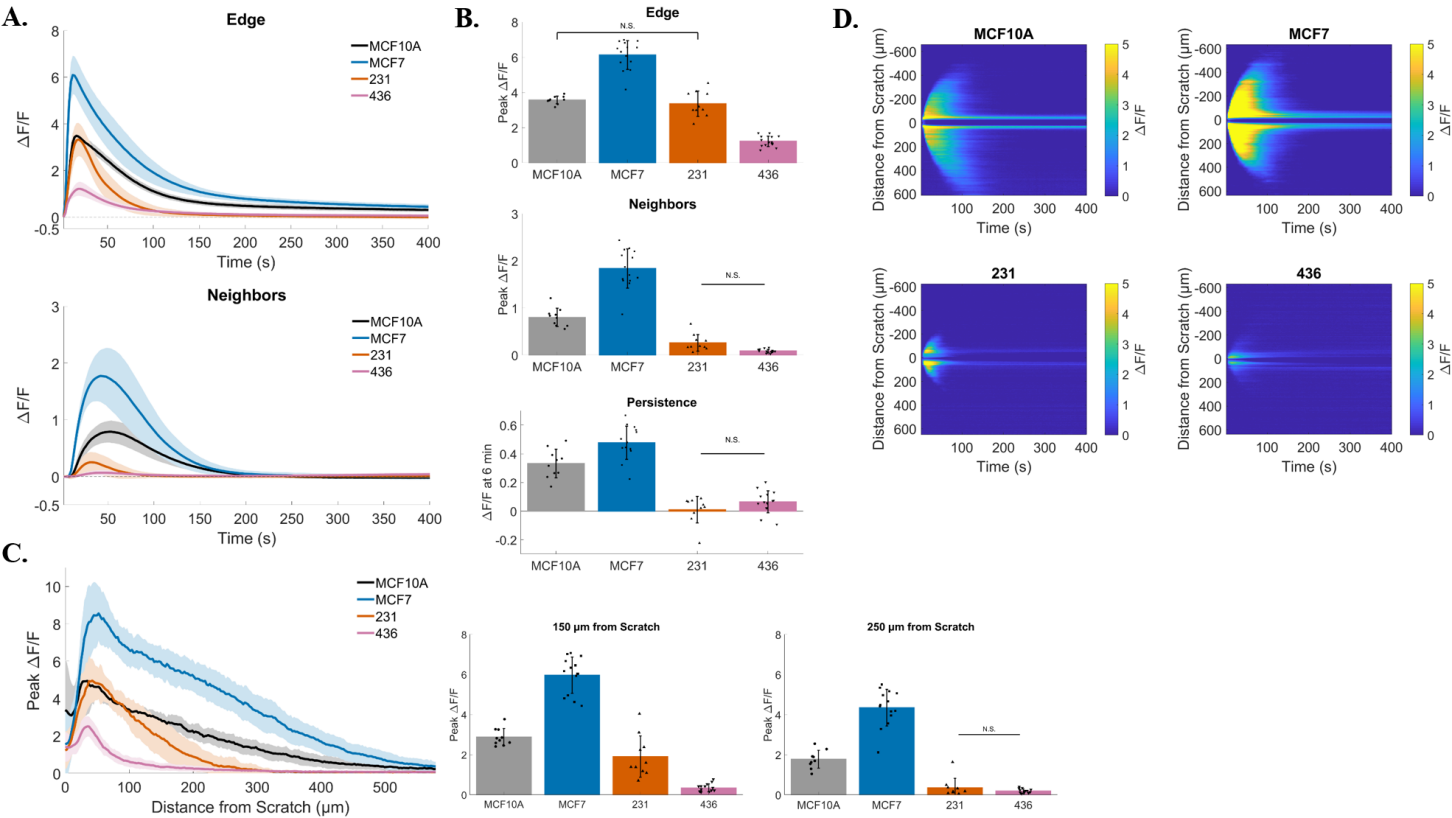
Signaling in response to scratch is disrupted in multiple breast cancer cell lines **(A)** Traces show ΔF/F plotted through time for wound edge cells (*upper traces*) *vs.* neighboring cells (*lower traces*) in human breast MCF10A epithelial cells and human breast MCF-7, MDA-MB-231, and MDA-MB-436 cancer cells. **(B)** Summary of peak ΔF/F analysis from wound edge cells and neighboring cells, and persistent calcium signaling at 6 minutes, for each cell line tested. Compared with non-tumorigenic breast epithelial cells (MCF10A), the three breast cancer cell lines showed significantly different signaling in response to scratch. MCF-7 cells showed an increased scratch-induced intracellular calcium in both wound edge and neighboring cells, as well as increased persistent calcium signaling. All of these parameters were significantly decreased in MDA-MB-231 and MDA-MB-436, compared with MCF10A (with exception of Edge peak ΔF/F MCF10A vs. 231). **(C)** Total distance of signal propagation away from the wound edge was plotted for each cell line tested. Traces and bar graphs show that signaling to neighbors is differentially propagated between cell lines. Compared with MCF10A, MDA-MB-231 and MDA-MB-436 cells show a diminished intercellular signaling between neighboring and wound edge cells, while this is enhanced in MCF-7 cells. **(D)** Kymographs (ROI-independent analysis) reveal similar mechanisms Kymographs (ROI-independent analysis) reveal similar mechanisms. Signaling across the cell monolayer in response to scratch is enhanced in MCF-7 cells compared with MCF10A, while it is severely reduced in MDA-MB-231 and MDA-MB-436 cells. Kymographs also show a reduced long term persistent signaling for MDA-MB-231 and MDA-MB-436 cells. Data presented as mean ± standard deviation. Unless noted by N.S., all values are significantly different with significance set at P < 0.05 via one-way ANOVA with a post-hoc Tukey's honest difference criterion. N.S. indicates no significant difference. Data represent N=10 (MCF10A), N=14 (MCF-7), N=10 (231), N=15 (436) in total from 2 independent experiments for each group.

In order to investigate if this response to a mechanical stimulus has broad significance in breast cancer, we tested our scratch assay in multiple cell lines and quantified the resulting calcium signaling. We plated human breast MCF10A epithelial cells and human breast MCF-7, MDA-MB-231, and MDA-MB-436 cancer cells to confluency, loaded them with Fluo-4 and quantified time lapse images of scratch-induced calcium (Figure 8). Compared with the non-tumorigenic breast epithelial cell line MCF10A, MCF-7 cells showed increased peak ΔF/F in wound edge cells (MCF10A vs. MCF-7: 3.6 ± 0.2 *vs.* 6.1 ± 0.8), peak ΔF/F in neighboring cells (MCF10A *vs.* MCF-7: 0.8 ± 0.2 *vs.* 1.8 ± 0.4), and long term persistence (ΔF/F at 6 min) (MCF10A *vs.* MCF-7: 0.3 ± 0.1 *vs.* 0.5 ± 0.1). Interestingly, signaling to neighboring cells and long term persistence was almost entirely blocked in MDA-MB-231 and MDA-MB-436 cells (Neighbors MCF10A *vs.* 231: 0.8 ± 0.2 *vs.* 0.26 ± 0.2. Persistence MCF10A *vs.* 231: 0.3 ± 0.1 *vs.* 0.01 ± 0.1) (Neighbors MCF10A *vs.* 436: 0.8 ± 0.2 *vs.* 0.08 ± 0.04. Persistence MCF10A *vs.* 436: 0.3 ± 0.1 *vs.* 0.06 ± 0.08). Peak signaling in edge cells was not significantly different between MCF10A and MDA-MB-231 cells (3.6 ± 0.2 *vs.* 3.3 ± 0.7, respectively), but reduced in MDA-MB-436 cells (3.6 ± 0.2 *vs.* 1.2 ± 0.3, respectively). Total distance of signal propagation away from the wound edge was then plotted for each cell line tested. In agreement with the measured peak ΔF/F for neighboring cells, the propagation of signal across the cell monolayer was enhanced with MCF-7, but diminished in MDA-MB-231 and MDA-MB-436 (Figure 8C and 8D). Traces and bar graphs show that signaling to neighbors is differentially propagated between cell lines, with MDA-MB-231 and MDA-MB-436 showing a reduced intercellular signaling between neighboring and wound edge cells (Figure 8C) [Peak ΔF/F at 150μm (MCF10A 2.9 ± 0.4, MCF-7 6.0 ± 0.9, 231 1.9 ± 1.0, 436 0.3 ± 0.2) and Peak ΔF/F at 250μm (MCF10A 1.8 ± 0.4, MCF-7 4.3 ± 0.9, 231 0.4 ± 0.5, 436 0.2 ± 0.1)].

### P2Y_2_ dependent signaling is conserved in breast cancer cells

**Figure 9:**
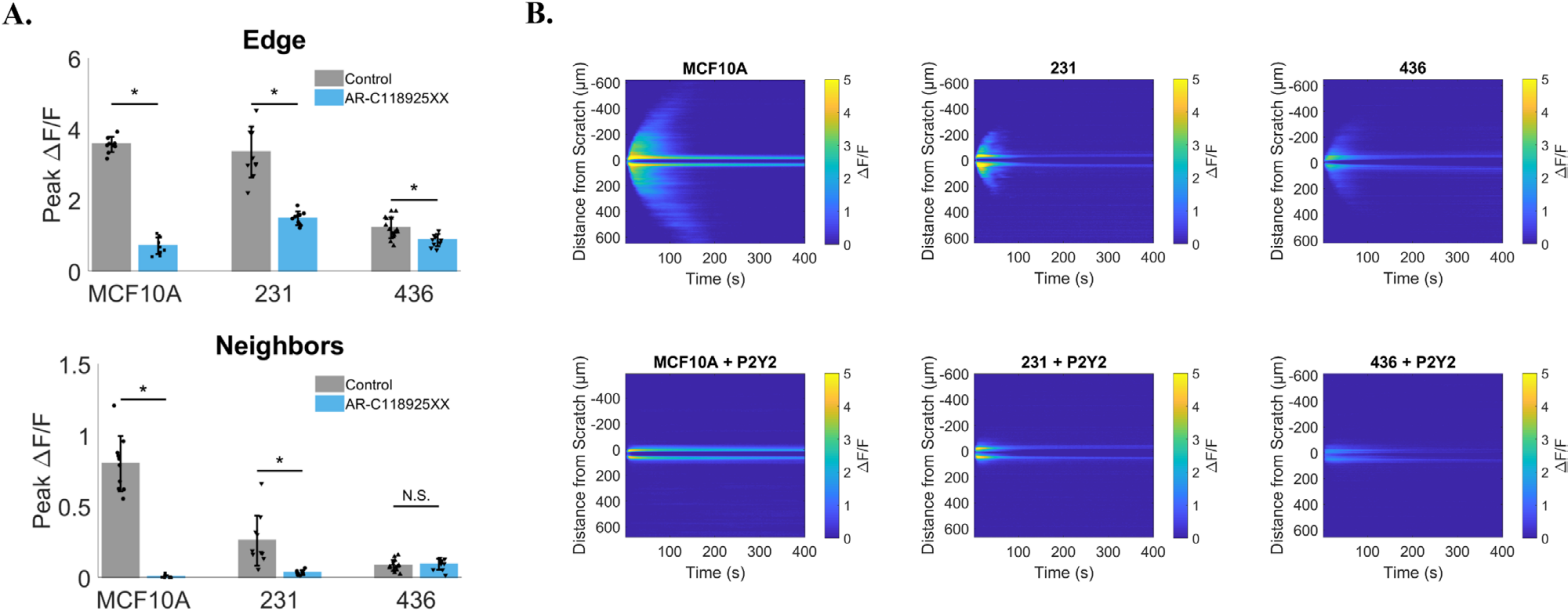
P2Y_2_ dependent signaling is conserved in breast cancer cells MCF10A, MDA-MB-231 and MDA-MB-436 cells were treated with a selective and competitive P2Y_2_ receptor antagonist (AR-C 118925XX) in order to test if scratch-induced signaling to neighboring cells was dependent on P2Y_2_. **(A)** Both MCF10A and MDA-MB-231 cells show a significant reduction in signaling to neighbors compared with control (signaling to neighbors in absence of AR-C 118925XX), suggesting a similar mechanism. Data show no significant difference in neighboring cells peak ΔF/F between control and P2Y_2_ inhibition for MDA-MB-436, however this is likely due to a lack of signaling to neighbors at baseline (control). **(B)** Kymographs (ROI-independent analysis) highlight inhibition of scratch-induced signaling to neighbors with selective P2Y_2_ antagonist. Data presented as mean ± standard deviation. ^*^indicates significance, P < 0.05 via paired t-test. N.S. indicates no significant difference. Data represent MCF10A (N=10 control and N=10 inhibitor), 231 (N=10 control and N=10 inhibitor), 436 (N=15 control and N=10 inhibitor) in total from 2 independent experiments for each group. Control data from Figure 8 was used for comparison here.

We assessed whether both normal epithelial breast cells and tumorigenic cells operated by similar mechanisms of signaling to neighboring cells to that of MCF-7 cells. We used an identical pharmacologic approach to block the P2Y_2_ receptor with the specific antagonist AR-C-118925XX (Figure 9) [Data presented as cell line vs. treatment with AR-C-118925XX: Peak ΔF/F at Edge (MCF10A: 3.6 ± 0.2 vs. 0.7 ± 0.2, 231 3.3 ± 0.7 vs. 1.5 ± 0.2, 436 1.2 ± 0.3 vs. 0.9 ± 0.2) and Peak ΔF/F at Neighbors (MCF10A: 0.8 ± 0.2 vs. 0.01 ± 0.01, 231 0.26 ± 0.2 vs. 0.03 ± 0.02, 436 0.08 ± 0.04 vs. 0.09 ± 0.04)]. We find that inhibition of P2Y_2_ receptors blocked calcium signal propagation to cells away from the wound edge in MCF10A and MDA-MB-231 cells, however there was no significant differences detected in MDA-MB-436 (Figure 9A). It is likely that differences weren't measurable in MDA-MB-436 cells due to the already diminished signaling between neighboring cells under untreated conditions. Interestingly, kymographs reveal, in some cases, that it is possible for MDA-MB-436 cells to show signaling to neighboring cells can occur (albeit drastically reduced) and that treatment with AR-C-118925XX can result in a complete inhibition of signaling away from the wound edge (Figure 9B).

## DISCUSSION

Our collective data describe the early mechanotransduction signaling events in response to scratch wounding in breast cancer cells. In human breast cancer cells, scratch wounding resulted in calcium signaling at the immediate wound edge followed by activation in distant cell neighbors, a spatiotemporal mechanism mediated by extracellular ATP signaling and P2Y_2_ receptors. Intercellular signaling was dependent on endoplasmic reticulum calcium stores, either internal or external calcium was sufficient for scratch-induced increases in cytosolic calcium in cells at the wound edge, and external calcium was necessary for persistent signaling at the wound edge. These findings establish the key early mechanisms of scratch-induced calcium signaling in breast cancer cells that could represent a variety of therapeutic points of entry to target potential oncogenic pathways spanning across diverse cellular events such as invasion, migration, cell cycle progression and cell death. Indeed, the reduction in signaling observed in cell lines that exhibit the most aggressive phenotypes (e.g. metastatic potential) not only suggest a selective advantage for inhibiting mechanically-activated signaling, but serve as a starting point for the design of therapeutic intervention in this pathway.

Signaling pathways and resulting cellular responses are often regulated by protein signaling complexes or enzymes, which are themselves controlled via post-translational modifications or varying conformational states. In contrast, calcium ions remain unaltered, yet are ubiquitous signaling molecules that regulate a variety of cellular processes across many tissue types. The specificity of calcium signaling to certain cellular pathways is thought to come from its characteristics; the cell can differentially respond to and initiate calcium signals of varying amplitude, sub-cellular location, persistence, and frequency (e.g. oscillations)[39–41]. For example, cell migration is dependent on rear-to-front calcium gradients [13, 15], cytosolic calcium oscillations drive invadopodia formation [42], and persistent cytosolic calcium or mitochondrial calcium overload can trigger necroptotic [43] or apoptotic [44] pathways. Calcium remodeling (i.e. changes in channel expression and/or directly measured calcium signaling) is observed in cancer cells and often correlates with oncogenic advantages (e.g. increases in metastatic potential or resistance to cell death)[14, 45]. This calcium-cancer relationship can even be extended to specific types of calcium signaling such as store-operated calcium entry, calcium oscillations, or sensitivity to ATP-stimulated calcium signaling [46]. The quantitative methods we have established here will provide the detail necessary to distinguish how breast cancer cells are responding to either transient P2Y_2_-dependent calcium signaling, or to persistently elevated calcium.

The advantage of scratch wound-dependent calcium signaling in cell monolayers is that it allows for observation and manipulation of population signaling. Moreover, this can be extrapolated to the tissue level and even directly observed *in vivo*[47]. The implications for tumor biology are clearly evident when considering the complexity of signaling in the tumor microenvironment, in which calcium may play an essential role [12]. In fact, methods for measuring calcium signaling in viable tumor slices have already been presented as proof of concept [48]. The connection between mechanical wounding in cell monolayers and calcium signaling has been previously shown in many cell types, including astrocytes [49, 50], hepatic cells [51], keratinocytes [52], endothelial cells [23], and various epithelial cells [22, 47, 53]. However, this has not been extensively characterized in mammary cells and to the best of our knowledge has only been briefly presented in single figure format from either mouse [54] or human [18] cells. Considering scratch wound-dependent calcium signaling in a broad sense across different epithelial cell types, the signaling mechanisms in mammary epithelial cells most closely resemble that of urothelial cells as described by Shabir and Southgate [55]. They described wound-dependent calcium signaling across cell monolayers, persistent calcium at the wound edge, and purinergic signaling. However, in many instances, they lacked sufficient quantification and selective inhibitors, which could explain the partial dependence of signal propagation to distant neighbors on both IP_3_ receptors and purinergic signaling (about 50% reduction when separately targeted pharmacologically). Aside from cell-type differences, what was particularly evident in our system was the reliable and complete inhibition of signal propagation when using pharmacologic intervention, which identified the P2Y_2_ receptor as an essential mechanism of intercellular communication. These easily quantifiable differences suggest a robust experimental system. In terms of cancer biology, this clear inhibition could also represent a dependence on specific signaling pathways for proliferation, survival, or metastasis, and would thus be an attractive therapeutic avenue. For example, published data suggest that *in vitro* cell migration (on the timescale of days) is dependent on extracellular calcium via the calcium sensing receptor in breast cancer [56], and in non-malignant keratinocytes migration (over 6-9 hours) requires mechanically activated ATP and P2Y-dependent calcium waves [52]. Finally, our novel approach to quantification using custom programming allowed us to rigorously test the early calcium signaling mechanisms that could underlie these long-term roles of calcium, but were not resolved with the longer time-frame of the previous studies.

What is particularly interesting is the connection between changes in calcium and EMT. EMT is a cell program that is involved in development and wound-healing, and results in the breakdown of cell junctions followed by increased cell proliferation, migration, and expression of stem cell markers. EMT is also thought to be a major component of breast cancer transformation, progression and drug resistance. Work linking EMT and changes in calcium in several breast cancer cell lines has been nicely reviewed by Azimi and Monteith [57]. For example, 1 hour chelation of intracellular calcium is able to block the ability of epidermal growth factor (EGF) to induce EMT cell modifications (measured 24 hours later) [18]. This could possibly be further linked to a specific amplitude in calcium signaling, since ATP-stimulated calcium resulted in a larger peak calcium but could not induce EMT markers such as vimentin [18]. Furthermore, changes in purinergic receptor-stimulated calcium signaling is associated with EMT and that targeting P2Y_6_[58] or P2X_5_[59] receptors reduced vimentin expression, induced by 48 hour hypoxia or 24 hour EGF stimulation respectively.

We applied our mechanical stimulus and in depth analysis to multiple breast cancer cell lines (MCF-7, MDA-MB-231, and MDA-MB-436) and compared them with an immortalized non-tumorigenic control cell line (MCF10A). Compared with MCF10A cells, we find that MCF-7 cells show an enhanced signal in the three main parameters measured: peak signaling in wound edge cells, peak signaling in neighboring cells, and long term persistence in wound edge cells. Conversely, we have observed a greatly reduced signaling in neighboring cells and long term persistence for MDA-MB-231 and MDA-MB-436 cells. This finding is significant because MDA-MB-231 and MDA-MB-436 cells are considered some of the most aggressive cell lines due to their increased capacity for tumor formation and ability to metastasize. Moreover, these cells have undergone permanent EMT, while MCF10A and MCF-7 have not. While calcium may play a role in the induction of EMT [18], its long term role may be less clear since mechanically-activated calcium is disrupted or lost in these cells as observed in the current study. Observed cancer cell phenotypes are typically a selective advantage for survival and drug resistance, and so what advantage this altered calcium signaling gives to these cells is of particular interest. One thought is that disruption of calcium signaling to neighboring cells may be why MDA-MB-231 and MDA-MB-436 cells are more metastatic. Intracellular calcium positively regulates cell to cell adhesion in fibroblasts [60], while metastasis requires the downregulation of cell to cell contacts with neighbors so that cells can migrate away. It is possible that this is achieved at the level of calcium and intracellular communication (e.g. ATP-induced calcium signaling).

The process of normalizing to baseline fluorescence (ΔF/F_0_) corrects for differences in sample-sample dye concentration, however there are some limitations. The expected result is that the fluorescence reflects the changes in intracellular calcium concentration. This approach relies on the assumption that cells within a sample (i.e. cells in a monolayer) homogeneously uptake the calcium sensitive dye Fluo-4. In addition, it is possible for variation of intracellular dye concentration to occur from sample to sample, despite that equal loading (molar amount and incubation duration) is controlled experimentally. This could be due to differences in a cell's ability to uptake the dye across the cell membrane, ability to trap the dye intracellularly and convert the dye to a calcium-sensitive form (i.e. expression of esterases), differences in dye leakage or clearance, dye compartmentalization or photobleaching due to repetitive exposure to the excitation source [61]. The ΔF/F_0_ normalization is a simple and efficient method for studying changes in calcium between experiments, but at the expense of neglecting information on the resting levels of intracellular calcium [62]. Differences in peak ΔF/F in any particular cell or region could be due to a variety of mechanisms, such as an increased release of calcium (e.g. increased expression of IP3 and/or RyR receptors or increased calcium concentrations), but ΔF/F cannot answer these mechanisms directly. Despite such limitations, we can still be confident that the spatial (spread to neighbors) and temporal (persistence) differences between cell lines are independent of issues with the dye. This is because it is very unlikely that the dye could load/convert specifically in certain areas over others within a sample and the extent of the Fluo-4 photobleaching is negligible in our conditions (Supplementary Figure 1).

The connections between mechanically-activated ATP signaling, purinergic receptors, calcium signaling, and EMT in *in vitro* cancer biology are mounting, but are still not well-defined. The real-time scratch assay established here provides comprehensive quantitative data, enabling identification of the molecular mechanisms that support rapid scratch-induced calcium signaling in breast cancer cells. These mechanisms now provide a clear framework for investigating which short-term calcium signals promote long-term changes in cancer cell biology.

## MATERIALS AND METHODS

### Cell culture

Human breast MCF10A epithelial cells and human breast MCF-7, MDA-MB-231, and MDA-MB-436 cancer cells were obtained from the American Type Culture Collection. Cells were maintained at 37°C, 5% CO_2_ in Dulbecco's Modified Eagle Medium (Corning, 10-017-CV) supplemented with 10% FBS (Atlantic Biologicals S11150H) and 1% penicillin-streptomycin (Gemini Bioproducts 400-109). In order to maintain stocks, cells were passaged using a brief wash in PBS (Quality biological, 114-058-101) followed by incubation with 0.25% Trypsin and 2.21mM EDTA (Corning, 25-053-CI) at 37°C, 5% CO_2_. For most experiments, cells were plated to confluency overnight in 35mm glass bottom microwell dishes (MatTek P35G-1.5-20-C). For a subset of experiments which required the separation of two confluent populations of cells in a single dish, cells were plated per manufacturer's instructions in culture insert dishes (Culture-insert 2 well, Ibidi, 80206)

### Reagents

Prior to imaging, cells were loaded using 4μM Fluo-4 AM (a cytosolic and calcium sensitive dye, Life Technologies, F14201) in Hanks Balanced Salt Solution containing calcium (HBSS+Ca^2+^, Gibco, 14025-092) for 30 minutes and washed in 1 mL HBSS+Ca^2+^ for 30 minutes. This final wash was used during imaging unless otherwise stated. To measure compromised cell plasma membranes, 1.5μM propidium iodide (PI, Sigma Aldrich, P4864) was added to the final wash step. For depletion of internal cellular calcium stores, cells were treated with 2μM thapsigargin (Sigma Aldrich, T9033) for 10 minutes prior to dye loading and washing. To deplete external calcium stores, dishes were washed twice briefly in 1 mL Hanks Balanced Salt Solution without calcium (HBSS-Ca^2+^, Gibco, 14025-092) supplemented with 100 μM EGTA (Sigma Aldrich, E3889) and then washed in a final 1mL HBSS-Ca^2+^ for 5 minutes prior to imaging. To deplete both internal and external calcium stores, cells were treated with thapsigargin, dye loaded, and washed prior to depleting external stores as described above. In order to replenish external calcium after depletion, cells were washed as described using HBSS-Ca^2+^, then during imaging 1mL HBSS-Ca^2+^ was aspirated followed by addition of 2mL HBSS+Ca^2+^. Apyrase (Sigma Aldrich, A6535) was reconstituted to 10 units/mL in HBSS+Ca^2+^ and used to cleave extracellular ATP to ADP/AMP. After dye loading and washing, dishes were incubated in 1 mL Apyrase (10units) for 10 minutes prior to scratch, without washout. A selective and competitive P2Y_2_ receptor antagonist (AR-C 118925XX, Tocris, 4890) was reconstituted to 10mM in DMSO and used at a 10μM working dilution. After dye loading and washing, dishes were incubated in 1 mL of 10μM 118925XX for 10 minutes prior to scratch, without washout. Different DMSO concentrations did not significantly affect calcium signaling (Supplementary Figure 5).

### Confocal imaging

Experiments were conducted on an Olympus IX81 microscope with a Fluoview FV1000 confocal laser scanning system. 512 × 512 pixel images with 2.485 μm/pixel spatial scale were collected at 2.0 μs sampling speed using a 10x objective and 0.4 numerical aperture. Potential dye activation due to laser excitation was measured using a 50 minute scan at 4 second frame rate (Supplementary Figure 1). Relative change in fluorescence (ΔF/F) was calculated across all time points and indicates no laser induced dye activation. In addition, propidium iodide (PI) was added to the media to identify membrane damage due to laser scanning during long periods of imaging. Images show no change in PI staining suggesting membrane damage is not occurring.

### Mechanical touch

Glass capillaries (Harvard Apparatus, 30-0053) were pulled and fire polished such that the end of the pipette tip was melted and sealed to a ~60μm rounded, bulbous end. Pipettes were attached to a motorized micromanipulator (Sutter Instrument, MP-225) used to maneuver the pipette in X, Y, and Z directions. Prior to mechanical touch of single cells, cells were brought into focus and the glass pipette was visualized using light microscopy in order to closely approach a single cell in a cell monolayer with the pipette. Then, 200 time series images with 2 second frame rate were collected using simultaneous DIC and Fluo-4 imaging. The pipette was then brought to the point of cell contact, as confirmed using visualization of pipette focus in DIC and initiation of Fluo-4 fluorescence. The pipette was then retracted for the remainder of the time series.

### Real-time scratch assay

Identical confocal settings and methods for generation and manipulation of glass pipettes were applied to both mechanical touch and scratch assays. As visualized in light microscopy, the glass pipette was used to compress the cell monolayer outside of the confocal scan area. Confocal time series imaging was immediately initiated (200 frames at 2 second frame rate) and the pipette was maneuvered in the X direction until a scratch was applied across the entirety of the cell monolayer in view. The pipette remained in its final place until completion of imaging. For experiments measuring long term calcium persistence 750 frames were collected at 4 second frame rate. For experiments using external calcium restoration after depletion 450 frames were collected at 4 second frame rate.

### ROI-based analysis

Relative change in Fluo-4 fluorescence was calculated as ΔF/F using regions of interest (ROI). Two approaches were implemented: manual and automated ROI generation. For manually-generated ROI analysis, ImageJ imaging software (NIH) and the Time Series Analyzer plugin (Version 3, https://imagej.nih.gov/ij/plugins/time-series.html) were used to set ROIs and calculate total pixel intensity. Three rectangular ROIs were set to encompass both the wound edge and neighboring cells outside of the wound edge. Two ROIs were selected from neighboring cells on either side of the scratch wound, and data were added to quantify fluorescence intensity in both directions from the wound edge. All ROI widths were set to 510 pixels, but due to variations in scratch size wound edge ROI height ranged from 50-70 pixels and ROI height from neighboring regions ranged from 200-230 pixels. ΔF/F was calculated by first subtracting background fluorescence and then dividing by F_0_, the fluorescence value before the scratch, for each data point. ΔF/F from cells at the scratch wound *vs.* neighboring cells were plotted in Microsoft Excel. Peak ΔF/F was determined by finding the maximum values and persistent ΔF/F was calculated using the value at frame 180 (360seconds/6 minutes) for each group. For long term persistence and calcium restoration experiments, only one ROI was used to measure ΔF/F at the wound edge over time. For the automated ROI analysis, custom scripts were written in MATLAB to automatically identify and analyze each ROI. For the set of experiments in which DIC images were acquired, the location of the glass pipette tip was extracted by creating binary images from the DIC images. The median location of this glass tip over time was used as the centerline of the scratch for creating each ROI. For the calcium restoration experiments, the scratch centerline was determined by finding the median location of the fluorescence minimum between the two calcium peaks on the edges of the scratch. In each case, a region centered on the scratch location with width 512 pixels (the image width) and height 100 pixels was used as the ROI for analysis of the edge. All pixels in the image not contained within the edge ROI were used for calculations describing the neighboring regions. For each ROI, the total intensity of pixels within the ROI was determined in each frame of the time lapse. This value was converted to ΔF/F by subtracting the value of the first frame from each frame and dividing the resulting subtracted value by the value of the first frame. Peak ΔF/F was determined by finding the maximum of this curve over time. The value of the ΔF/F curve at 6 minutes (frame 180) was used as a measure of fluorescence persistence.

### ROI-independent analysis

A ROI-independent analytical approach was used to investigate the distance the fluorescence signal traveled away from the scratch in which the value of ΔF/F was calculated for each distance from the scratch in the image (equivalent to the calculations described above if ROIs 1 pixel in height covered the entire image). The peak value of ΔF/F was calculated for each of these curves and plotted as function of distance from the scratch. We present the values of this curve at two particular distances, 60 pixels (150μm) and 201 pixels (500μm) away from the scratch centerline in bar graph format to show that the signal propagates to different distances in distinct conditions. A second ROI-independent analysis used to analyze both distance and persistence was based on the generation of kymographs in MATLAB, which were converted to binary images from which distance and persistence were quantified (Figure 2E). For each scratch assay, intensity profiles were computed by taking the average pixel intensity along the axis of the scratch wound (image width) for each point along the y-axis (image height, or perpendicular to the scratch wound) at each time point (200 frames total). The F_0_ y-axis intensity profile was computed using the average along the x-axis for the first frame. Kymographs were then computed by taking the normalized y-axis profile (ΔF/F) at each time point and displayed graphically [i.e. lining them up left (t=0 s) to right (t = 400 s)]. Kymographs were then processed to binary images which were computed by thresholding each kymograph at 0.48. This threshold level was determined by first computing the automatic global threshold level (MATLAB's ‘greythresh’ function) for each vehicle treated scratch assay and then taking the average threshold level across all assays. The binary images were used to quantify the total distance that the fluorescence signal traveled away from the scratch to neighboring cells (termed maximum distance of propagation) and the total time of persisting calcium signal at the wound edge (termed maximum time of persistence). Maximum distance of calcium propagation was computed by taking the average of the two maximum height values (i.e. furthest distance where fluorescence was collected from above and below the wound edge) of the binary image (Figure 2E, top and bottom blue lines). The maximum time of persistent calcium was computed by taking the average of the two maximum length values (i.e. maximum time frame where fluorescence was collected along the wound edge) from the binary image (Figure 2E, top and bottom pink lines).

### Statistics

Statistical analyses were performed using MATLAB. A paired t-test was used when comparing single conditions: MCF-7 Edge *vs.* Neighbors (Figure 2), Control *vs.* 0Ca^2+^ + EGTA (Figure 6), Control *vs.* AR-C-118925XX within each cell line (Figure 9), and significance was set at P < 0.05. For comparison between multiple conditions: Control, thapsigargin, 0Ca^2+^ + EGTA, thapsigargin/0Ca^2+^ + EGTA (Figure 5); Control, Apyrase, AR-C-118925XX (Figure 7); MCF10A, MCF-7, MDA-MB-231, MDA-MB-436) (Figure 8), a one-way ANOVA with a post-hoc Tukey's honest difference criterion was used and significance set at P < 0.05.

## SUPPLEMENTARY MATERIALS FIGURES AND VIDEOS



















## Abbreviations

ECM: Extracellular matrix
EMT: Epithelial-to-mesenchymal transition
ATP: Adenosine triphosphate
ADP: Adenosine diphosphate
AMP: Adenosine monophosphate
UT: Uridine triphosphate
FBS: Fetal bovine serum
PBS: Phosphate buffered saline
EDTA: Ethylenediaminetetraacetic acid
HBSS: Hanks Balanced Salt Solution containing calcium
PI: Propidium iodide
EGTA: Ethylene glycol-bis(β-aminoethyl ether)-N,N,N',N'-tetraacetic acid
DMSO: Dimethyl sulfoxide
DIC: Differential interference contrast
ROI: Region of interest
ΔF/F: Relative change in fluorescence
SERCA: Sarco-endoplasmic calcium ATPase
0Ca^2+^ + EGTA: Calcium-depleted media
P2Y_2_: Purinergic receptor Y-type isoform 2

## References

[1] Kai F, Laklai H, Weaver VM (2016). Force Matters: Biomechanical Regulation of Cell Invasion and Migration in Disease. Trends Cell Biol.

[2] Wei SC, Yang J (2016). Forcing through Tumor Metastasis: The Interplay between Tissue Rigidity and Epithelial-Mesenchymal Transition. Trends Cell Biol.

[3] Butcher DT, Alliston T, Weaver VM (2009). A tense situation: forcing tumour progression. Nat Rev Cancer.

[4] Majeski HE, Yang J (2016). The 2016 John J. Abel Award Lecture: Targeting the Mechanical Microenvironment in Cancer. Mol Pharmacol.

[5] Boyd NF, Guo H, Martin LJ, Sun L, Stone J, Fishell E, Jong RA, Hislop G, Chiarelli A, Minkin S, Yaffe MJ (2007). Mammographic density and the risk and detection of breast cancer. N Engl J Med.

[6] Pollán M, Ascunce N, Ederra M, Murillo A, Erdozáin N, Alés-Martínez J, Pastor-Barriuso R (2013). Mammographic density and risk of breast cancer according to tumor characteristics and mode of detection: a Spanish population-based casecontrol study. Breast Cancer Res.

[7] Paszek MJ, Zahir N, Johnson KR, Lakins JN, Rozenberg GI, Gefen A, Reinhart-King CA, Margulies SS, Dembo M, Boettiger D, Hammer DA, Weaver VM (2005). Tensional homeostasis and the malignant phenotype. Cancer Cell.

[8] Wozniak MA, Desai R, Solski PA, Der CJ, Keely PJ (2003). ROCK-generated contractility regulates breast epithelial cell differentiation in response to the physical properties of a three-dimensional collagen matrix. J Cell Biol.

[9] Wozniak MA, Chen CS (2009). Mechanotransduction in development: a growing role for contractility. Nat Rev Mol Cell Biol.

[10] Nguyen-Ngoc KV, Cheung KJ, Brenot A, Shamir ER, Gray RS, Hines WC, Yaswen P, Werb Z, Ewald AJ (2012). ECM microenvironment regulates collective migration and local dissemination in normal and malignant mammary epithelium. Proc Natl Acad Sci USA.

[11] Hack CC, Stoll MJ, Jud SM, Heusinger K, Adler W, Haeberle L, Ganslandt T, Heindl F, Schulz-Wendtland R, Cavallaro A, Uder M, Beckmann MW, Fasching PA, Bayer CM (2017). Correlation of mammographic density and serum calcium levels in patients with primary breast cancer. Cancer Med.

[12] Monteith GR, Prevarskaya N, Roberts-Thomson SJ (2017). The calcium-cancer signalling nexus. Nat Rev Cancer.

[13] Prevarskaya N, Skryma R, Shuba Y (2011). Calcium in tumour metastasis: new roles for known actors. Nat Rev Cancer.

[14] Marchi S, Pinton P (2016). Alterations of calcium homeostasis in cancer cells. Curr Opin Pharmacol.

[15] Iamshanova O, Fiorio Pla A, Prevarskaya N (2017). Molecular mechanisms of tumour invasion: regulation by calcium signals. J Physiol.

[16] Cui C, Merritt R, Fu L, Pan Z (2017). Targeting calcium signaling in cancer therapy. Acta Pharm Sin B.

[17] Wei SC, Fattet L, Tsai JH, Guo Y, Pai VH, Majeski HE, Chen AC, Sah RL, Taylor SS, Engler AJ, Yang J (2015). Matrix stiffness drives epithelial-mesenchymal transition and tumour metastasis through a TWIST1-G3BP2 mechanotransduction pathway. Nat Cell Biol.

[18] Davis FM, Azimi I, Faville RA, Peters AA, Jalink K, Putney JW, Goodhill GJ, Thompson EW, Roberts-Thomson SJ, Monteith GR (2014). Induction of epithelial-mesenchymal transition (EMT) in breast cancer cells is calcium signal dependent. Oncogene.

[19] Schäfer M, Werner S (2008). Cancer as an overhealing wound: an old hypothesis revisited. Nat Rev Mol Cell Biol.

[20] Balkwill F, Charles KA, Mantovani A (2005). Smoldering and polarized inflammation in the initiation and promotion of malignant disease. Cancer Cell.

[21] de Visser KE, Eichten A, Coussens LM (2006). Paradoxical roles of the immune system during cancer development. Nat Rev Cancer.

[22] Hinman LE, Beilman GJ, Groehler KE, Sammak PJ (1997). Wound-induced calcium waves in alveolar type II cells. Am J Physiol.

[23] Chifflet S, Justet C, Hernández JA, Nin V, Escande C, Benech JC (2012). Early and late calcium waves during wound healing in corneal endothelial cells. Wound Repair Regen.

[24] Justet C, Hernández JA, Torriglia A, Chifflet S (2016). Fast calcium wave inhibits excessive apoptosis during epithelial wound healing. Cell Tissue Res.

[25] Hanahan D, Weinberg RA (2011). Hallmarks of cancer: the next generation. Cell.

[26] Prevarskaya N, Skryma R, Shuba Y (2010). Ion channels and the hallmarks of cancer. Trends Mol Med.

[27] Frame MK, de Feijter AW (1997). Propagation of mechanically induced intercellular calcium waves via gap junctions and ATP receptors in rat liver epithelial cells. Exp Cell Res.

[28] Abu Khamidakh AE, Juuti-Uusitalo K, Larsson K, Skottman H, Hyttinen J (2013). Intercellular Ca(2+) wave propagation in human retinal pigment epithelium cells induced by mechanical stimulation. Exp Eye Res.

[29] Furuya K, Enomoto K (1990). Real-time imaging of intracellular calcium change with simultaneous single channel recording in mammary epithelial cells. Brain Res Bull.

[30] Enomoto K, Furuya K, Yamagishi S, Maeno T (1992). Mechanically induced electrical and intracellular calcium responses in normal and cancerous mammary cells. Cell Calcium.

[31] Furuya K, Enomoto K, Yamagishi S (1993). Spontaneous calcium oscillations and mechanically and chemically induced calcium responses in mammary epithelial cells. Pflugers Arch.

[32] Enomoto K, Furuya K, Yamagishi S, Maeno T (1993). Proliferationassociated increase in sensitivity of mammary epithelial cells to inositol-1,4,5-trisphosphate. Cell Biochem Funct.

[33] Enomoto K, Furuya K, Yamagishi S, Oka T, Maeno T (1994). The increase in the intracellular Ca2+ concentration induced by mechanical stimulation is propagated via release of pyrophosphorylated nucleotides in mammary epithelial cells. Pflugers Arch.

[34] Homolya L, Steinberg TH, Boucher RC (2000). Cell to cell communication in response to mechanical stress via bilateral release of ATP and UTP in polarized epithelia. J Cell Biol.

[35] Yin J, Xu K, Zhang J, Kumar A, Yu FS (2007). Wound-induced ATP release and EGF receptor activation in epithelial cells. J Cell Sci.

[36] Burnstock G (2006). Pathophysiology and therapeutic potential of purinergic signaling. Pharmacol Rev.

[37] Zhang JL, Liu Y, Yang H, Zhang HQ, Tian XX, Fang WG (2017). ATP-P2Y2-β-catenin axis promotes cell invasion in breast cancer cells. Cancer Sci.

[38] Rafehi M, Burbiel JC, Attah IY, Abdelrahman A, Müller CE (2017). Synthesis, characterization, and *in vitro* evaluation of the selective P2Y2 receptor antagonist AR-C118925. Purinergic Signal.

[39] Samanta K, Parekh AB (2017). Spatial Ca2+ profiling: decrypting the universal cytosolic Ca2+ oscillation. J Physiol.

[40] Berridge MJ (1997). The AM and FM of calcium signalling. Nature.

[41] Berridge MJ, Bootman MD, Roderick HL (2003). Calcium signalling: dynamics, homeostasis and remodelling. Nat Rev Mol Cell Biol.

[42] Sun J, Lu F, He H, Shen J, Messina J, Mathew R, Wang D, Sarnaik AA, Chang WC, Kim M, Cheng H, Yang S (2014). STIM1- and Orai1-mediated Ca(2+) oscillation orchestrates invadopodium formation and melanoma invasion. J Cell Biol.

[43] Nomura M, Ueno A, Saga K, Fukuzawa M, Kaneda Y (2014). Accumulation of cytosolic calcium induces necroptotic cell death in human neuroblastoma. Cancer Res.

[44] Orrenius S, Zhivotovsky B, Nicotera P (2003). Regulation of cell death: the calcium-apoptosis link. Nat Rev Mol Cell Biol.

[45] Azimi I, Roberts-Thomson SJ, Monteith GR (2014). Calcium influx pathways in breast cancer: opportunities for pharmacological intervention. Br J Pharmacol.

[46] Stewart TA, Yapa KT, Monteith GR (2015). Altered calcium signaling in cancer cells. Biochim Biophys Acta.

[47] Aihara E, Hentz CL, Korman AM, Perry NP, Prasad V, Shull GE, Montrose MH (2013). *In vivo* epithelial wound repair requires mobilization of endogenous intracellular and extracellular calcium. J Biol Chem.

[48] Koh J, Hogue JA, Sosa JA (2016). A Novel Ex Vivo Method for Visualizing Live-Cell Calcium Response Behavior in Intact Human Tumors. PLoS One.

[49] Gao K, Wang CR, Jiang F, Wong AY, Su N, Jiang JH, Chai RC, Vatcher G, Teng J, Chen J, Jiang YW, Yu AC (2013). Traumatic scratch injury in astrocytes triggers calcium influx to activate the JNK/c-Jun/AP-1 pathway and switch on GFAP expression. Glia.

[50] Kraft A, Jubal ER, von Laer R, Döring C, Rocha A, Grebbin M, Zenke M, Kettenmann H, Stroh A, Momma S (2017). Astrocytic Calcium Waves Signal Brain Injury to Neural Stem and Progenitor Cells. Stem Cell Reports.

[51] Sung YJ, Sung Z, Ho CL, Lin MT, Wang JS, Yang SC, Chen YJ, Lin CH (2003). Intercellular calcium waves mediate preferential cell growth toward the wound edge in polarized hepatic cells. Exp Cell Res.

[52] Takada H, Furuya K, Sokabe M (2014). Mechanosensitive ATP release from hemichannels and Ca2+ influx through TRPC6 accelerate wound closure in keratinocytes. J Cell Sci.

[53] Appleby PA, Shabir S, Southgate J, Walker D (2015). Sources of variability in cytosolic calcium transients triggered by stimulation of homogeneous uro-epithelial cell monolayers. J R Soc Interface.

[54] Sammak PJ, Hinman LE, Tran PO, Sjaastad MD, Machen TE (1997). How do injured cells communicate with the surviving cell monolayer?. J Cell Sci.

[55] Shabir S, Southgate J (2008). Calcium signalling in woundresponsive normal human urothelial cell monolayers. Cell Calcium.

[56] Saidak Z, Boudot C, Abdoune R, Petit L, Brazier M, Mentaverri R, Kamel S (2009). Extracellular calcium promotes the migration of breast cancer cells through the activation of the calcium sensing receptor. Exp Cell Res.

[57] Azimi I, Monteith GR (2016). Plasma membrane ion channels and epithelial to mesenchymal transition in cancer cells. Endocr Relat Cancer.

[58] Azimi I, Beilby H, Davis FM, Marcial DL, Kenny PA, Thompson EW, Roberts-Thomson SJ, Monteith GR (2016). Altered purinergic receptor-Ca2+ signaling associated with hypoxia-induced epithelial-mesenchymal transition in breast cancer cells. Mol Oncol.

[59] Davis FM, Kenny PA, Soo ET, van Denderen BJ, Thompson EW, Cabot PJ, Parat MO, Roberts-Thomson SJ, Monteith GR (2011). Remodeling of purinergic receptor-mediated Ca2+ signaling as a consequence of EGF-induced epithelialmesenchymal transition in breast cancer cells. PLoS One.

[60] Ko KS, Arora PD, Bhide V, Chen A, McCulloch CA (2001). Cell-cell adhesion in human fibroblasts requires calcium signaling. J Cell Sci.

[61] Bruton JD, Cheng AJ, Westerblad H (2012). Methods to detect Ca(2+) in living cells. Adv Exp Med Biol.

[62] Rudolf R, Mongillo M, Rizzuto R, Pozzan T (2003). Looking forward to seeing calcium. Nat Rev Mol Cell Biol.

