# Supplementary material for: Real-time scratch assay reveals mechanisms of early calcium signaling in breast cancer cells in response to wounding
Source: Oncotarget. 2018 May 18;9(38):25008–24. doi: 10.18632/oncotarget.25186 (PMC5982755; doi:10.18632/oncotarget.25186)
Supplement: Supplementary file 1 [file oncotarget-09-25008-s001.pdf]

Real-time scratch assay reveals mechanisms of early calcium signaling in breast cancer cells in response to wounding

SUPPLEMENTARY MATERIALS

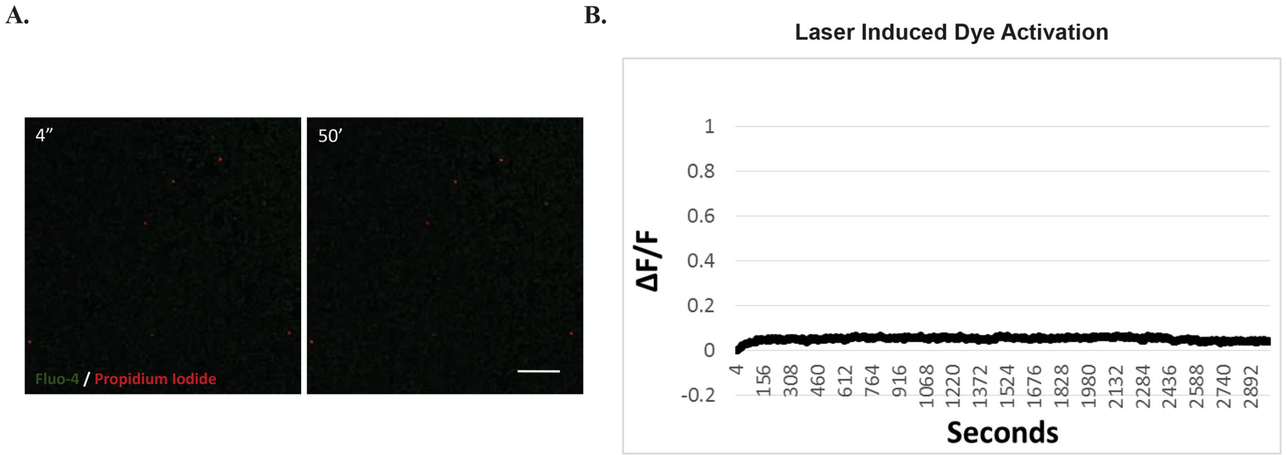

Supplementary Figure 1: Confocal laser scanning does not activate Fluo-4 or damage cell membranes during 50 minute scans. (A) Human breast MCF-7 cancer cells were loaded with the calcium sensitive dye Fluo-4 and treated with propidium iodide (PI, a membrane impermeable nuclear stain) which fluoresces only when it is able to cross compromised cellular membranes and bind DNA. Cells were then imaged for 50 minutes at a 4 second frame rate to determine if long term laser excitation was damaging to cells. PI staining indicates no change between 4 seconds and 50 minutes, suggesting cells are not being damaged. **(B)** In addition, ΔF/F was calculated across the time series to measure potential dye activation from laser excitation. The data show that baseline ΔF/F values are not altered by laser excitation and confirming the absence of photodamage by our imaging protocol.

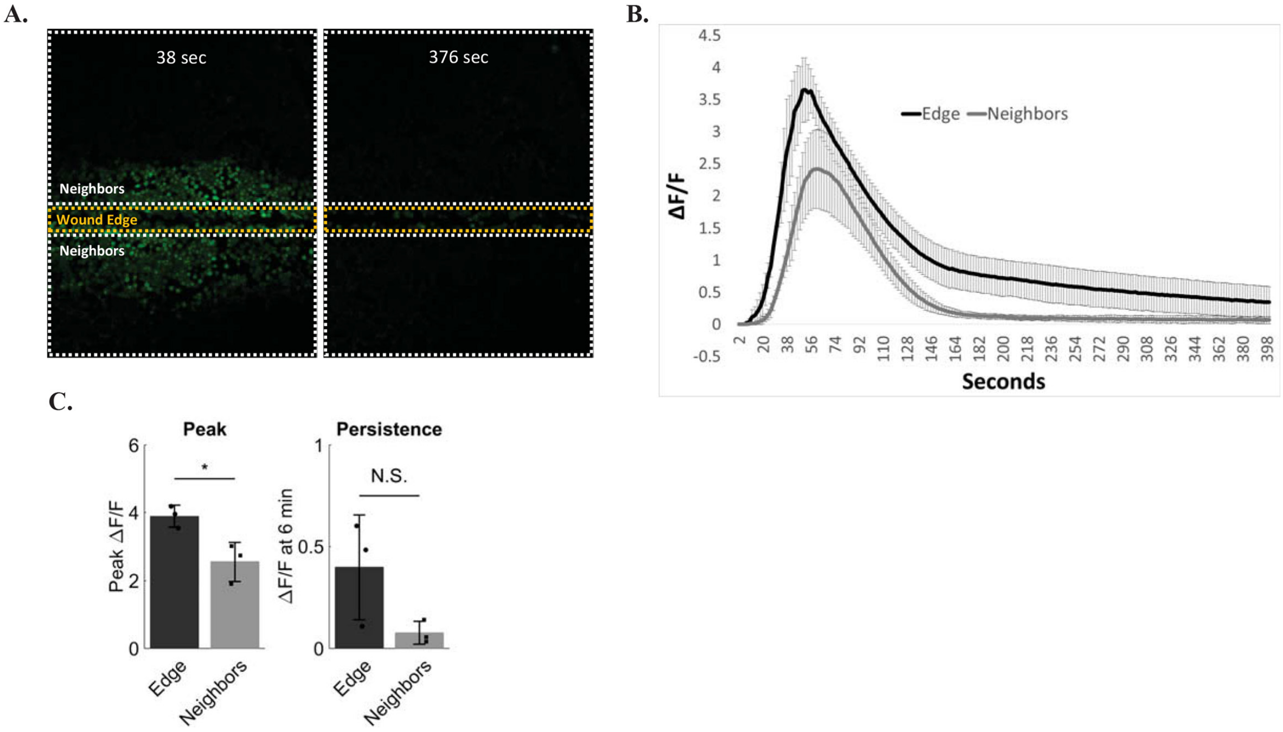

Supplementary Figure 2: Manually selected ROI-based analysis. For manually-generated ROI analysis, ImageJ imaging software and the Time Series Analyzer plugin were used to set ROIs and calculate total pixel intensity. **(A)** Three rectangular ROIs were set to encompass both the wound edge and neighboring cells outside of the wound edge. All ROI widths were set to 510 pixels, but due to variations in scratch size wound edge ROI height ranged from 50-70 pixels and ROI height from neighboring regions ranged from 200-230 pixels. **(B)** Scratch assay data (ΔF/F) from cells at the wound edge and neighboring cells were plotted up to 400 seconds. **(C)** Peak (maximum) and persistent [value at frame 180 (360seconds/6 minutes)] ΔF/F was calculated for each group. Edge: 3.9 ± 0.3 for peak, 0.4 ± 0.3 for persistence. Neighbors: 2.5 ± 0.6 for peak, 0.1 ± 0.1 for persistence. Data presented as mean ± standard deviation. Data represent N=3. *indicates significance from edge, $P < 0.05$ via paired t-test.

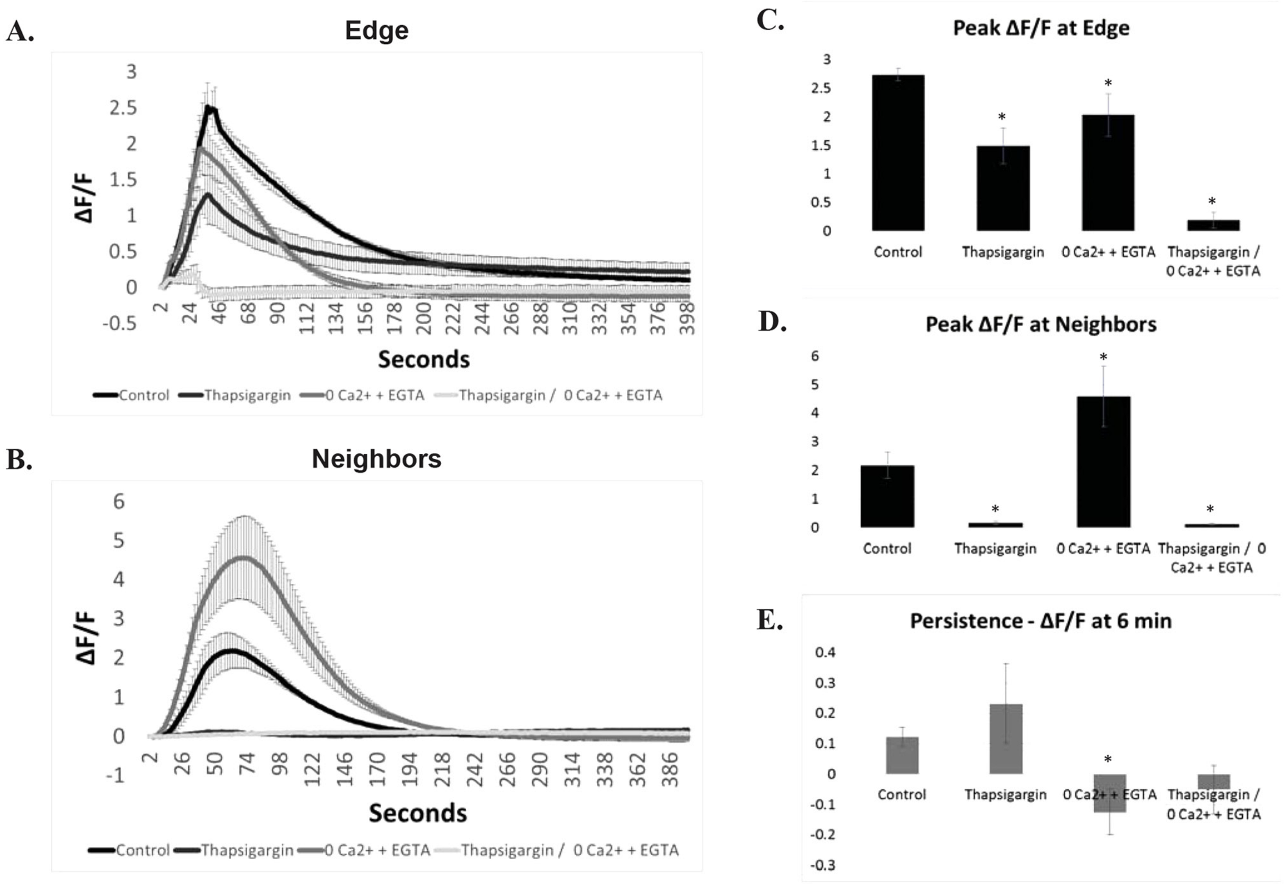

Supplementary Figure 3: Manually selected ROI-based quantification zero calcium and thapsigargin treated groups. Scratch assay data (ΔF/F) from cells at the wound edge **(A)** and neighboring cells **(B)** were plotted up to 400 seconds and show traces for control, thapsigargin, $0Ca^{2+}$ + EGTA, and thapsigargin/$0Ca^{2+}$ + EGTA treated groups. **(C)** Peak ΔF/F for cells at the wound edge [control: 2.7 ± 0.1, thapsigargin: 1.5 ± 0.3, $0Ca^{2+}$ + EGTA: 2.0 ± 0.4, and thapsigargin/$0Ca^{2+}$ + EGTA: 0.2 ± 0.1]. **(D)** Peak ΔF/F for neighboring cells [control: 2.2 ± 0.5, thapsigargin: 0.2 ± 0.1, $0Ca^{2+}$ + EGTA: 4.6 ± 1.1, and thapsigargin/$0Ca^{2+}$ + EGTA: 0.1 ± 0.1]. **(E)** Persistence at the wound edge (ΔF/F at frame 360, 6 minutes) [control: 0.12 ± 0.03, thapsigargin: 0.23 ± 0.13, $0Ca^{2+}$ + EGTA: -0.12 ± 0.08, and thapsigargin/$0Ca^{2+}$ + EGTA: -0.05 ± 0.08]. Data presented as mean ± standard deviation. Data represent N=3 for control and N=4 for thapsigargin, $0Ca^{2+}$ + EGTA, and thapsigargin/$0Ca^{2+}$ + EGTA treated groups. *indicates significance from control, $P < 0.05$ via one-way ANOVA with a post-hoc Tukey's honest difference criterion.

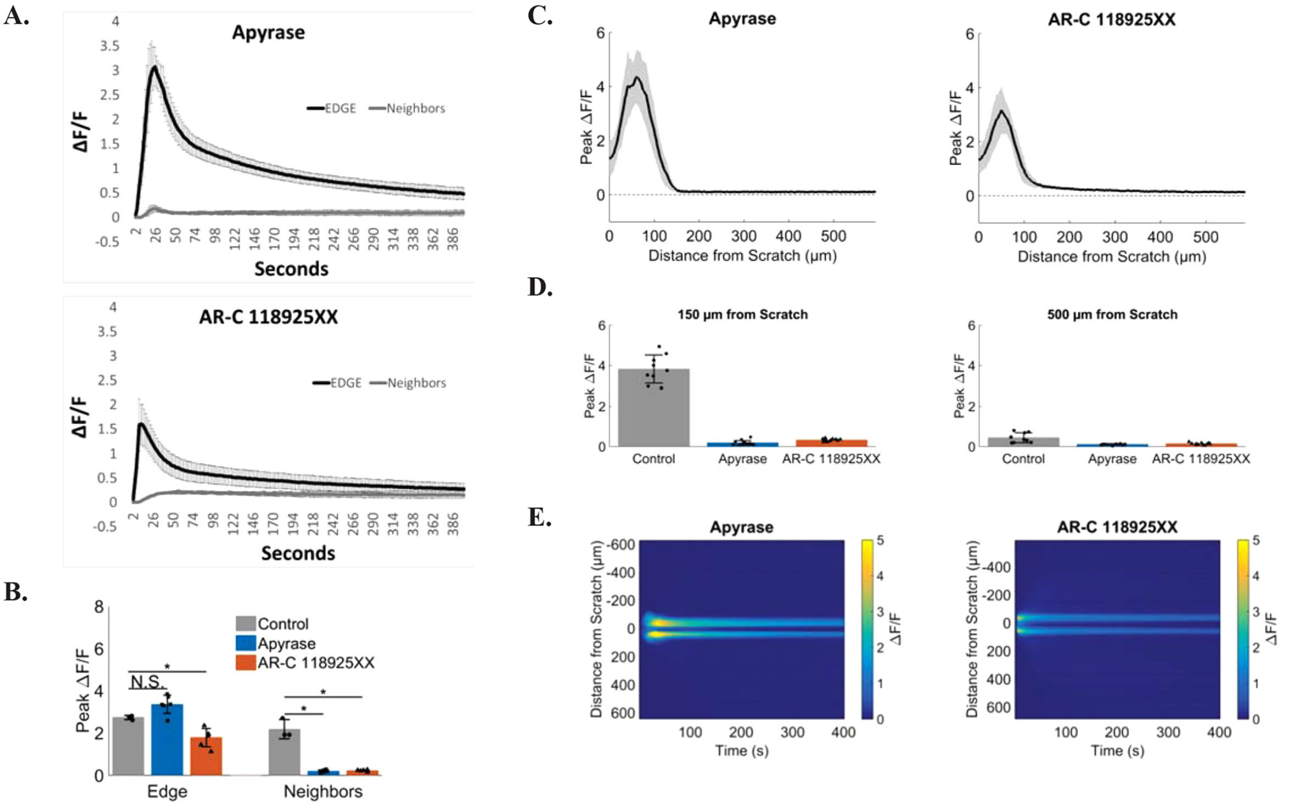

Supplementary Figure 4: Manually selected ROI-based quantification and distance analysis of Apyrase and $P2Y_2$. **(A)** Scratch assay data (ΔF/F) from cells at the wound edge and neighboring cells were plotted up to 400 seconds and show traces for Apyrase and AR-C118925XX treated groups. **(B)** Peak ΔF/F for cells at the wound edge [Apyrase: 3.4 ± 0.4, AR-C118925XX: 1.8 ± 0.4]. Peak ΔF/F for neighboring cells [Apyrase: 0.2 ± 0.1, AR-C118925XX: 0.2 ± 0.1]. Data presented as mean ± standard deviation. Data represent N=6 for each group. **(C, D)** Peak ΔF/F was calculated for distances at 150μm and 500μm from the edge of the scratch and also plotted to show total distance of signal propagation to neighboring cells away from the wound edge. For both Apyrase and AR-C118925XX treated groups, peak ΔF/F was minimal close to the wound edge (Apyrase: 0.2 ± 0.1 at 150μm, AR-C118925XX: 0.3 ± 0.1 at 150μm) and also at far distances (Apyrase: 0.1 ± 0.03 at 500μm, AR-C118925XX: 0.1 ± 0.1 at 500μm). **(E)** Kymographs were generated using y-axis time projections at each time point through 400 seconds for Apyrase and AR-C118925XX treated groups. Kymographs show a lack of signal propagation away from the wound edge, but show persistent calcium at the edge. Data presented as mean ± standard deviation. Data represent N=12 for each group. *indicates significance from edge, $P < 0.05$ via paired t-test.

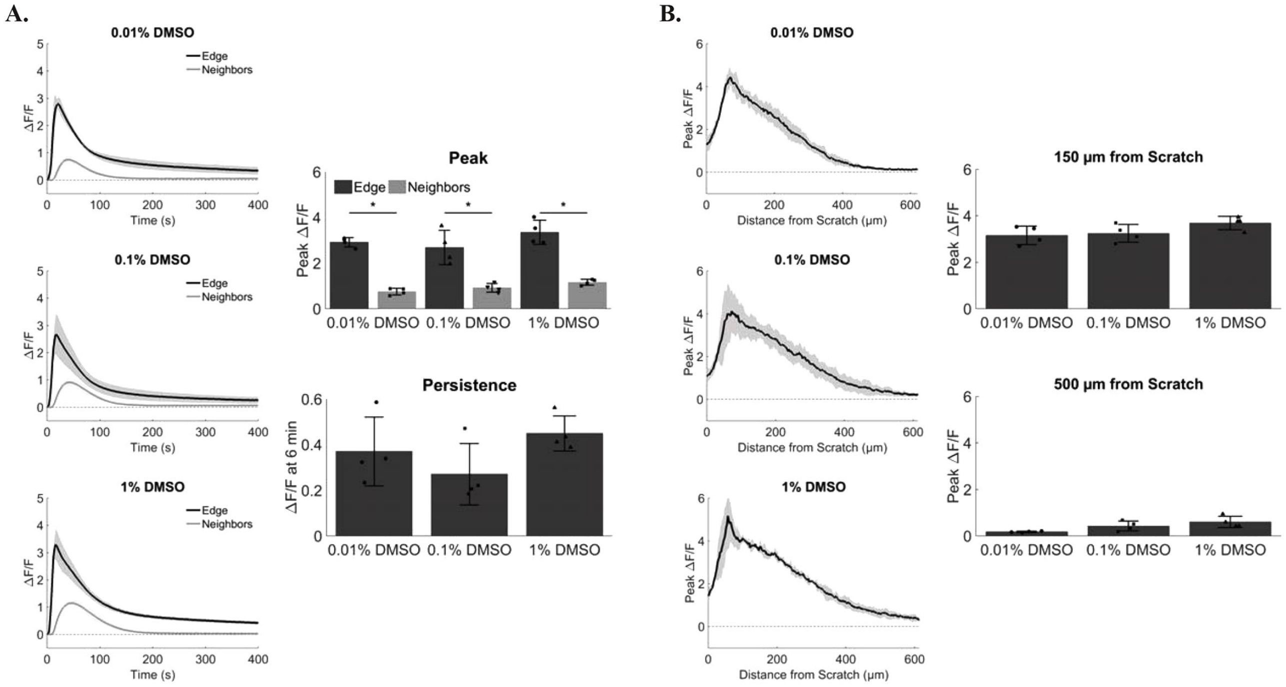

Supplementary Figure 5: Automated ROI-based quantification and distance analysis of 0.01%, 0.1%, 1% DMSO controls. **(A)** Scratch assay data (ΔF/F) from cells at the wound edge and neighboring cells were plotted up to 400 seconds and show traces for 0.01%, 0.1%, 1% DMSO treated controls. Peak ΔF/F for cells at the wound edge [0.01%: 2.9 ± 0.2, 0.1%: 2.7 ± 0.7, 1%: 3.3 ± 0.5]. Peak ΔF/F for neighboring cells [0.01%: 0.8 ± 0.1, 0.1%: 0.9 ± 0.2, 1%: 1.2 ± 0.1]. Persistence at the wound edge (ΔF/F at frame 360, 6 minutes) [0.01%: 0.4 ± 0.2, 0.1%: 0.3 ± 0.1, 1%: 0.5 ± 0.1]. **(B)** Peak ΔF/F was calculated for distances at 150μm and 500μm from the edge of the scratch and also plotted to show total distance of signal propagation to neighboring cells away from the wound edge. Peak ΔF/F at 150μm [0.01%: 3.2 ± 0.4, 0.1%: 3.2 ± 0.4, 1%: 3.7 ± 0.3]. Peak ΔF/F at 500μm [0.01%: 0.2 ± 0.03, 0.1%: 0.4± 0.2, 1%: 0.6 ± 0.2]. Data presented as mean ± standard deviation. Data represent N=3 for each group. *indicates significance from edge, $P < 0.05$ via paired t-test.

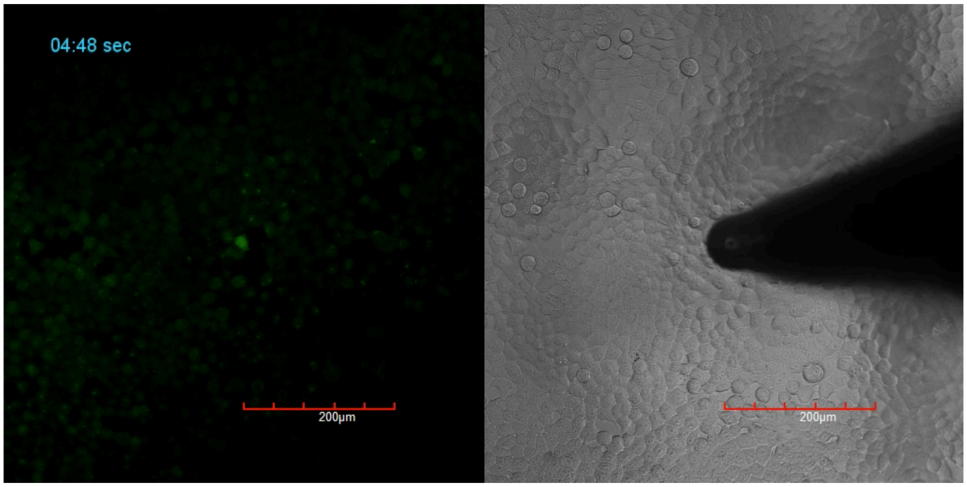

Supplementary Video 1: Mechanical Touch. Human breast MCF-7 cancer cells were loaded with the calcium sensitive dye Fluo-4 and mechanically stimulated using a blunt fire-polished glass microprobe.

See Supplementary File 1

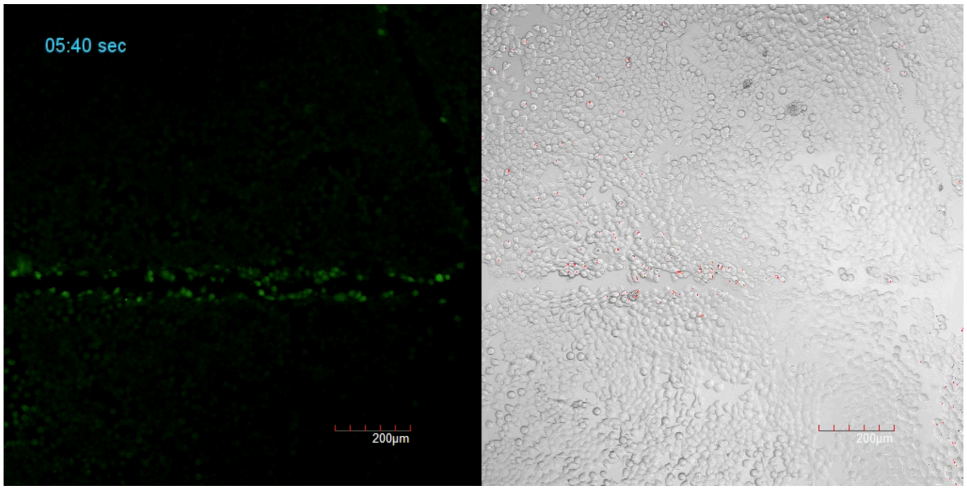

Supplementary Video 2: Real Time Scratch Assay. Fluo-4 loaded MCF-7 cell monolayers were simultaneously scratched with a glass pipette and imaged for 400 seconds.

See Supplementary File 2

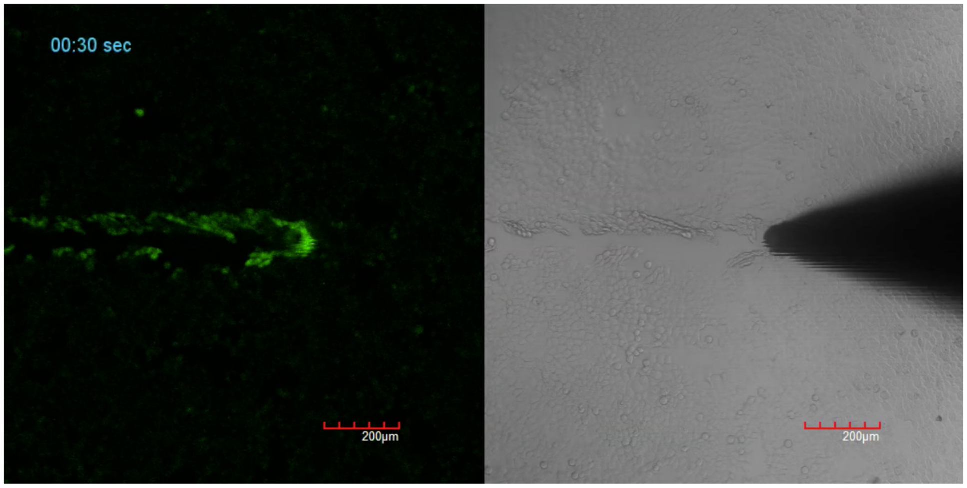

Supplementary Video 3: Thapsigargin. Thapsigargin treated cells (depletion of intracellular calcium) failed to propagate calcium signaling away from the wound edge.

See Supplementary File 3

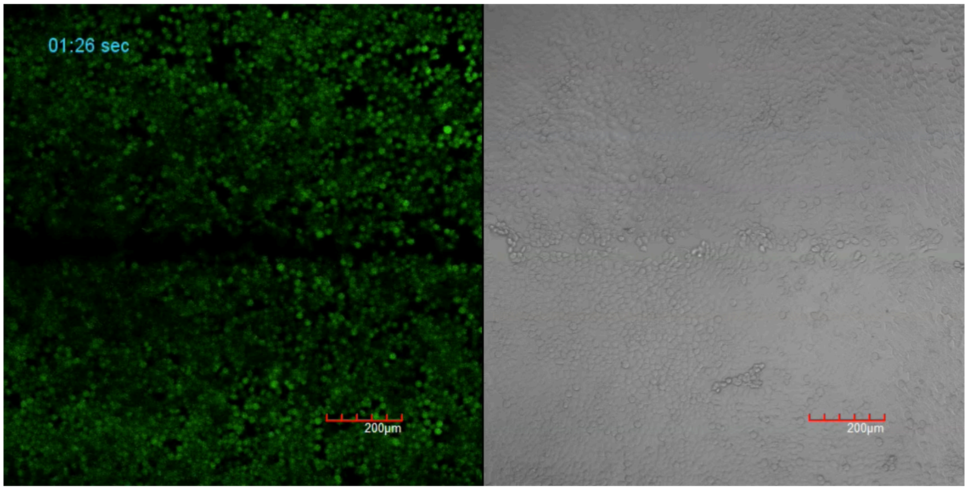

Supplementary Video 4: 0Ca2+ + EGTA. Depletion of extracellular calcium stores (0Ca^{2+} + EGTA) did result in signal propagation to neighboring cells but blocked persistent calcium at the wound edge.

See Supplementary File 4

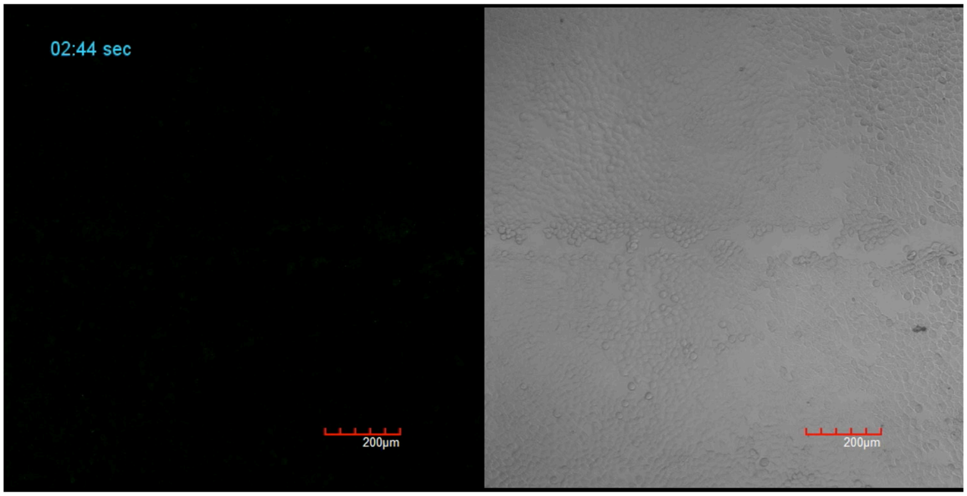

Supplementary Video 5: Thapsigargin/0Ca2+ + EGTA. Depletion of both intracellular and extracellular calcium (Thapsigargin/0Ca^{2+} + EGTA) entirely blocked calcium signaling.

See Supplementary File 5

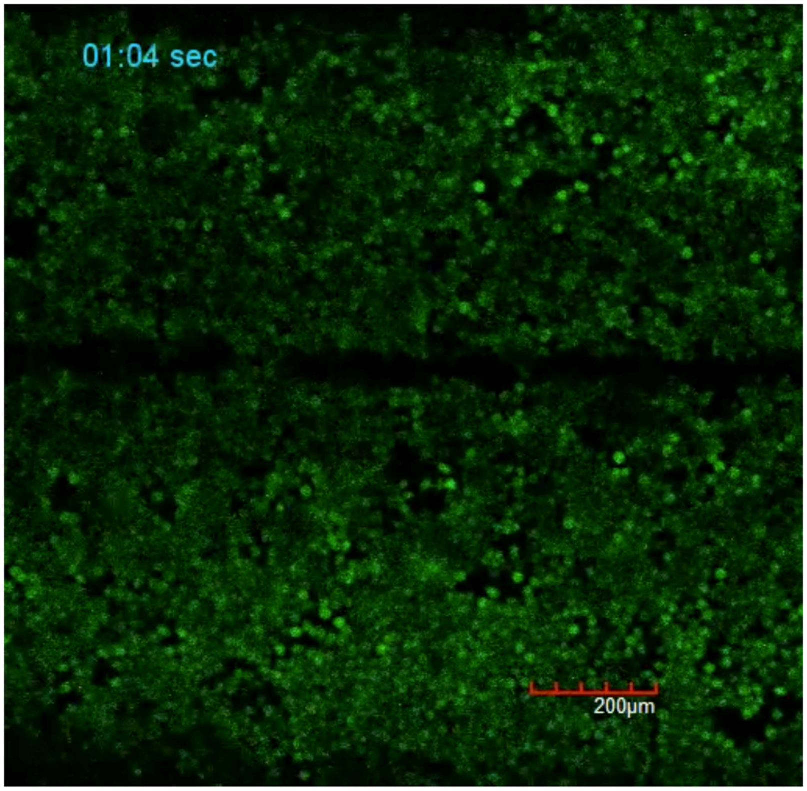

Supplementary Video 6: Calcium Restoration After Depletion. Cells were incubated in $0Ca^{2+}$ + EGTA media, calcium was then replenished in the external media which resulted in a sudden and lasting rescue of persistent calcium in cells at the wound edge.

See Supplementary File 6

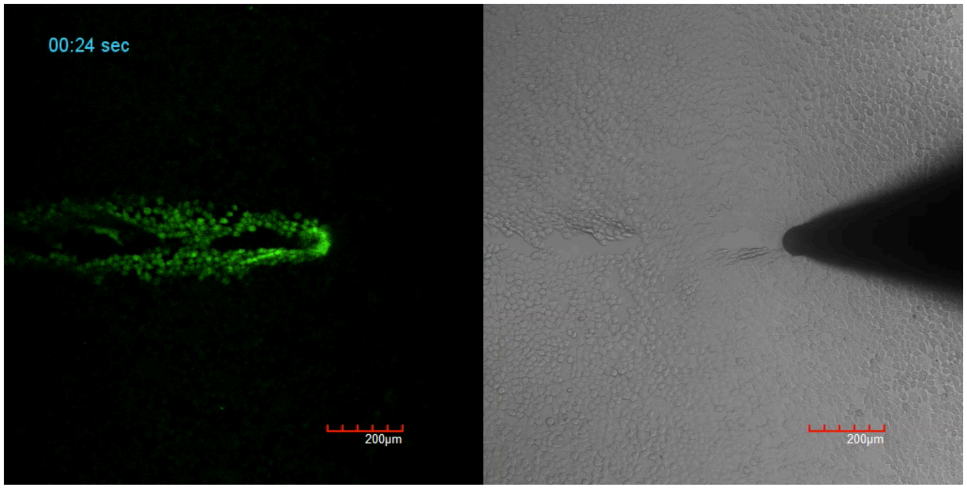

Supplementary Video 7: Apyrase. Cells were treated with Apyrase in order to cleave extracellular ATP to ADP/AMP which blocked signal propagation to neighboring cells.

See Supplementary File 7

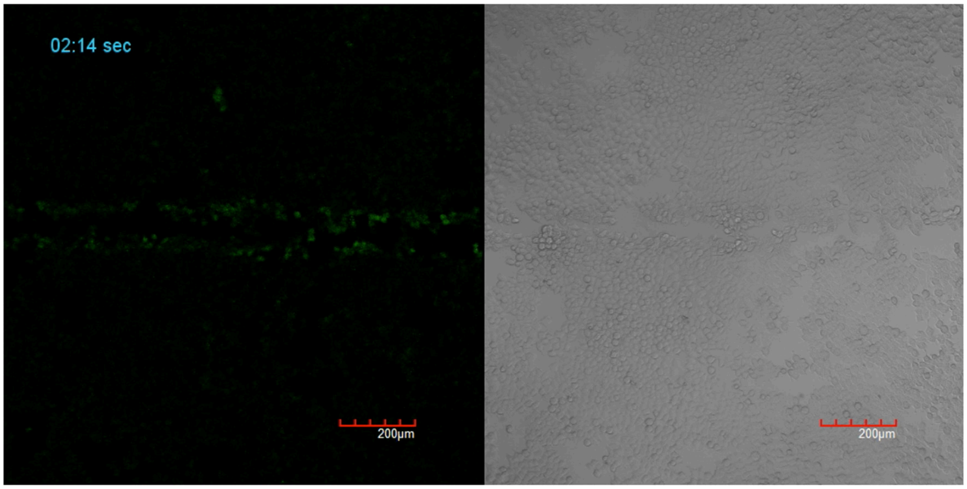

Supplementary Video 8: P2Y2 Antagonist. Cells were treated with a selective $P2Y_2$ receptor antagonist which blocked signal propagation to neighboring cells.

See Supplementary File 8
